# Associations between snowboard coaches teaching interaction, motion guidance, and effectiveness assessment competencies and learners intentions to continue lessons

**DOI:** 10.3389/fpsyg.2025.1633094

**Published:** 2025-09-23

**Authors:** Junchen Liu, Tianhai Hu, Jiachen Yin

**Affiliations:** ^1^Hubei University, School of Physical Education, Hubei, China; ^2^Blizzard Sports Academy, Livingston, NJ, United States; ^3^Huazhong University of Science and Technology, School of Architecture and Urban Planning, Wuhan, Hubei, China

**Keywords:** Chinese snowboard education, coach-learner interaction, teaching interaction and feedback, motion guidance and optimization, effectiveness assessment and customization, loyalty, intention to continue lessons

## Abstract

**Background:**

The rise in winter sports following the 2022 Beijing Winter Olympics has led to a surge in snowboarding enthusiasts. However, domestic snowboard coach training remains at a rudimentary stage, resulting in substandard instructor competencies that create negative customer experiences and seriously impede the development of China's snowboarding industry.

**Purpose:**

To address these coaching quality challenges, this study developed a three-dimensional pedagogical model and examined how snowboard coaches' teaching competencies influence participants' loyalty, operationalized as intention to continue lessons.

**Methods:**

Using a mixed methods design, the study collected survey responses from 250 recreational snowboarders at two major ski resorts in China, supplemented by on-site coaching observations. Quantitative analyses, including correlation, ordinary least squares (OLS) regression, and quantile regression, were employed to examine the relationship between the proposed three-dimensional coaching model and participants' loyalty.

**Results:**

Factor analysis refined the traditional interpersonal-technical-pedagogical framework into three dimensions: Teaching Interaction and Feedback (TIF), Motion Guidance and Optimization (MGO), and Effectiveness Assessment and Customization (EAC). Together with overall satisfaction, these competencies showed significant positive associations with loyalty (*R*^2^ = 0.754). Quantile regression revealed heterogeneous effects across loyalty levels: TIF and MGO exerted the strongest influence at lower loyalty levels, EAC became more salient toward median-upper quantiles, and overall satisfaction had notable effects only at the very lowest loyalty levels.

**Conclusion:**

Snowboard coaches' competencies function as an interdependent system that supports learners psychological needs in distinct ways across different engagement levels. Teaching interaction and motion guidance are most critical for establishing a supportive climate and skill clarity in the early stages, while effectiveness assessment becomes increasingly important as learners seek personalized progression. Overall satisfaction plays a disproportionately important role at the lowest loyalty levels, underscoring the need to reduce onboarding frictions and strengthen perceptions of safety. These findings refine Self-Determination Theory applications in outdoor sport education and highlight that adaptive, segment-aware coaching strategies are essential for promoting retention and sustainable participation in winter sports.

## 1 Introduction

The successful hosting of the 2022 Beijing Winter Olympics has served as a powerful catalyst, significantly propelling the leapfrog development of China's winter sports and ice-snow industry (Peng et al., 2024; JIANG et al., 2022). According to the China Mass Winter Sports Consumption Market Research Report released by the General Administration of Sports, during the 2023–2024 winter season, 26 national ski resorts received 26.085 million visitors, marking a 91. 77% increase year over year, while generating integrated cultural-sports-tourism revenue of 19.349 billion yuan, representing 139.95% growth compared to the previous season (General Administration of Sport of China, 2024b). Beyond these popular national resorts, winter sports economies in low-latitude southern alpine regions have shown accelerated development (Li and Jie, 1 20). 2024 statistics reveal that central, southern and southwestern China host 65, 15, and 47 ski resorts, respectively (General Administration of Sport of China, 2024a). This rapid expansion in southern regions has not only fostered north-south synergy in winter sports, but also improved public participation throughout the country (Yu et al., 2024; TANG et al., 2022; Lei et al., 2022).

However, this rapid expansion has brought numerous challenges to China's snowboarding industry (Ma et al., 2023; Li et al., 2016; Wei et al., 2025), with the most serious being the critical shortage of qualified snowboard coaches Wu and Liu (2020). Industry reports indicate a 70% deficit in professional coaching personnel (General Administration of Sport of China, 2017), forcing many snowboard schools to rely on uncertified 'wild coaches'. In some cases, novice enthusiasts have paid high fees for instruction only to receive incorrect basic training (e.g., improper boot fitting Li and Yuqin, 2023) from these unqualified coaches. Even coaches with basic national certification often fall short of international teaching standards, underscoring a talent gap that threatens the customer experience and sustainable development of the snow sports sector.

A key underlying issue has been the traditional emphasis of Chinas coach training programs on technical skills at the expense of pedagogical skills (Light et al., 2014). In contrast, leading international programs (e.g. the American Association of Snowboard coaches) stress a balance of **Technical competence**, **Interpersonal Competence**, and **Pedagogical Competence** (PSIA-AASI, 2025). The disparity is evident in practice: many Chinese coaches excel in demonstrating techniques but lack the soft skills and adaptive teaching strategies needed to engage and guide diverse learners (das Neves Salles et al., 2025). Deficiencies in communication and motivational skills hinder effective coach—learner interaction (Shelley et al., 2010), while a drill-based segmented teaching approach (rather than an integrated coaching strategy) can impede the holistic understanding of snowboarding skills of learners (China National Vocational Qualification Training Program for Social Sports Instructors, 2023). Furthermore, the predominant seasonal model of the “migratory coach”,^1^ in which southern Chinese ski resorts hire temporary coaches during the peak season, undermines continuity and consistency of instruction (Tide News, 2023). Together, these issues in coach preparation and deployment have led to inconsistent coaching quality on slopes. Empirical evidence shows that these instructional shortcomings directly diminish learners experiences and retention (Richards and Gordon, 2017; Moravecz et al., 2025). In fact, a substantial proportion of first-time trainees disengage from formal lessons due to suboptimal learning outcomes–including slow skill acquisition, poor technique development, and lack of confidence progression (Lin et al., 2024)—often resorting to self-teaching or peer instruction despite the greater risks of inefficient learning and injury (Han and Jia, 2023; Zhang et al., 2023; Meng et al., 2025). This deterioration in coaching standards—where inexperienced learners often end up instructing other beginners due to instructor shortages (China News Service, 2025)—threatens to perpetuate poor technique acquisition and unsafe practices in the snowboard instruction market, ultimately restricting snowboard development and long-term industry growth (Sun et al., 2023). Addressing the coach training gap is therefore critical for improving learner loyalty (intention to continue lessons) and development in snow sports programs (Galvan et al., 2012).

In response to these challenges, the present study focuses on enhancing the education of snowboard coaches through a pedagogically oriented training model (Goodyear et al., 2013). Drawing on best practices from international frameworks and the literature on sport pedagogy, three broad domains of coach competence were identified as critical: technical competence (i.e., precise skill demonstration and safe movement instruction), interpersonal competence (i.e., effective communication, motivation, and engagement) and pedagogical competence (i.e. structured lesson planning and adaptive teaching methods) (Demiral and Nazıroğlu, 2024). By integrating these domains into an improved training curriculum for snowboard instructors, the study aimed to strengthen coaches ability to foster fun learning experiences and to encourage learners **loyalty (intention to continue lessons)** in recreational snowboard lessons (Gray et al., 2018).

Specifically, this research investigated how an improved coach education program—focusing on technical, interpersonal, and pedagogical development—would affect the experiences of learners in a real-world leisure sport context. Veteran snowboard instructors collaborated to redesign the curriculum, which was then applied in select resorts for feedback from learners. Unlike prior PE studies on service quality and participation intention, this study (i) examines an under-researched snowboard context in Chinas emerging resorts with acute coach shortages, (ii) operationalizes an SDT-aligned tri-competency curriculum with a validated measurement scale, and (iii) evaluates intention to continue lessons in live resort delivery, including distributional (quantile) effects. Of particular interest was whether and to what extent this pedagogy-focused approach would improve the overall learning atmosphere, skill acquisition, and **loyalty**. By examining these questions, the study contributes to a broader understanding of coach development within physical education and sport pedagogy, highlighting how targeted instructional strategies can promote both immediate skill gains and longer-term lesson retention (Koh et al., 2017).

## 2 Literature review

### 2.1 Enhancing technical, pedagogical, and interpersonal competencies in coach education

Based on mature snowboard coach training programs (PSIA-AASI, 2025) and the latest research in sport pedagogy, this study aims to systematically improve the three-dimensional competencies of snowboard coaches, namely, technical, pedagogical, and interpersonal skills. Specifically, considering that technical skills involve detailed movements and precise demonstrations, the study refers to the American Association of Snowboard Instructors (AASI) curriculum to refine and supplement the technical training component within the Chinese snowboard coach education. Meanwhile, improvements in pedagogical and interpersonal competencies will be achieved by reviewing and synthesizing prominent academic findings, thus developing an optimized pedagogical model tailored to the Chinese context. To provide a theoretical foundation for understanding how these coaching competencies influence learner motivation and continued participation, this study draws upon Self-Determination Theory.

**Self-determination theory (SDT)** represents a significant framework within motivational psychology (Weiner, 1990). Unlike traditional approaches that conceptualize motivation as a singular construct, SDT distinguishes between autonomous and controlled forms of motivation (Deci and Ryan, 2008). The theoretical foundation of SDT emerged from research conducted in the 1970s, with formal development occurring primarily during the 1980s through the work of Deci and Ryan (1980, 2013). Subsequently, both the theoretical framework and its practical applications have undergone substantial expansion (Adams et al., 2017; Ryan and Deci, 2024).

Within physical education, SDT research shows that teachers pedagogical design and interpersonal style are the chief levers through which basic psychological needs (autonomy, competence, relatedness) are supported or thwarted (Behzadnia, 2021a; Behzadnia et al., 2018). Need-supportive (vs. controlling) behaviors reliably enhance need satisfaction, autonomous motivation, engagement, and well-being, with students causality orientations shaping how they interpret the same behaviors (Behzadnia, 2021a; Behzadnia et al., 2018). Interventions that deliberately combine pedagogy with interpersonal-skill training—e.g., the coach-as-youth-development-specialist program—improve coaches capacity for personal/social development alongside technical instruction (Ettl Rodŕıguez et al., 2023). Longitudinal work in youth basketball further shows that coaches interpersonal and pedagogical skills mediate enjoyment, perceived competence, and sustained participation, independent of win-loss records (Morales-Belando et al., 2023), and communication training on sensitive topics (e.g., menstruation) strengthens the coach-athlete relationship (Bergström et al., 2023). Collectively, this literature motivates a dual-competency view: effective instruction couples sound pedagogical design with need-supportive interpersonal communication, which underpins our focus on coach education.

Extending SDT beyond school-based PE, work in outdoor, adventure, and lifestyle sports shows that a coachs interpersonal style and pedagogical planning are pivotal for satisfying autonomy, competence, and relatedness under hyperdynamic, risk-mediated conditions (Collins and Collins, 2022; Leeder and Beaumont, 2025; Collins and Brymer, 2020). In adventure contexts, coaches adopt an “it-depends” decision-making approach, where richer situational awareness enables better calibration of strategies to performer and environment (Collins and Collins 2022). Lifestyle and nature-sport settings further highlight values, inclusion, and participant-focused facilitation, calling for adaptable, flexible, and culturally sensitive coaching practices rather than purely competition-driven models (Leeder and Beaumont, 2025; Collins and Brymer, 2020). Translating these insights to snowboard instruction, model-based lesson design for beginners (e.g., progressive task structuring, feedback loops) provides a scaffold that converts environmental variability into learning opportunities (Zhang and Chaetnalao, 2024), while beginner-oriented snowsport studies underscore the importance of safety literacy and high-quality coach-learner interaction for engagement and persistence (Siqi and Chaetnalao, 2024). Professional development pathways (e.g., supervised practice and continuing education) help instructors enact need-supportive communication—clear rationales, calibrated choice, and scaffolded challenge—alongside sound pedagogy, aligning SDTs dual-competency template with resort-based snowboard education (Pighetti, 2021).

Therefore, based on the findings of previous research, the present study specifically addresses these competency gaps by advocating for a pedagogical approach that emphasizes creative instructional activities, regular assessment, and personalized training strategies to enhance teaching skills. In addition, interpersonal competencies are improved through reflective and embodied learning practices, paying close attention to fulfilling learners' basic psychological needs, and fostering attentive listening and reciprocal communication between coaches and learners. This integrative approach is expected to significantly increase the motivation, satisfaction, and long-term **loyalty** of learners in snowboard lessons.

### 2.2 Intention to continue lessons in snowboard instruction

In the context of coach education and sport pedagogy, the intention learners to continue lessons–conceptualized in commercial settings as the analogous “repurchase intention”—represents a critical psychological construct that bridges instructional quality with long-term learner engagement (Wu et al., 2015; Hennig-Thurau et al., 2001). In this study, **loyalty** is used as the primary outcome term, and the term “repurchase intention” is used only when referring to the consumer-behavior literature that informs this educational analogue. While originally rooted in marketing and consumer behavior literature (Hellier et al., 2003; Mittal and Kamakura, 2001), this concept holds particular relevance in educational and athletic contexts, where “continuing lessons” parallels a learner's psychological commitment to pursue further instruction (Cope et al., 2022; Greene, 1973; Chaves-Castro et al., 2025). This behavioral intention reflects not only satisfaction with current experiences but also the perceived value of ongoing skill development and the quality of coach—learner relationships.

In physical activity programs, various factors have been shown to influence lesson continuance intentions and broader participation (Behnam et al., 2021; Allen and Shaw, 2009). Howat and Assaker (Kranzinger et al., 2024) highlight the potential for immersion and a supportive environment to increase the willingness of the learner to continue training. Similarly, Yoshida et al. (2023) document how well-designed curricula and service quality enhance the commitment to educational sports programs, while Chaves-Castro et al. (2025) highlight that in outdoor sports events, sustainable practices and event quality contribute to participants desire to return. In line with this perspective, Shelley et al. (2010) found that need-supportive teaching and students autonomous orientation fostered greater intention to remain active in college PE, whereas need-thwarting behaviors lowered students desire to continue. Teixeira et al. (2022) found that the alignment between exercise intensity and individual preferences significantly influenced participants' enjoyment, exercise habits, and continued participation intention, suggesting the importance of tailoring activity intensity to match participant preferences in sustaining engagement.

Snowboarding, in particular, has rapidly emerged as a major focus for leisure participation, making the willingness to continue taking lessons (or “reenroll”) an increasingly important metric for programs seeking to develop lifelong engagement. Past research has identified factors such as enjoyment (Shen, 2016) and service quality (Zhou and Zhang, 2024) as significant predictors of a learners decision to continue lessons. Grant (Grant, 2013) shows that personal background and previous experiences, such as ease of equipment rental or instructor communication, strongly affect whether individuals return for further instruction. However, most of these studies focus on well-established snowboard destinations and robust instructor education systems (Zhou and Zhang, 2024; Shen, 2016; Grant, 2013). Accordingly, our study focuses on novice/recreational learners enrolled in commercial snowboard lessons—rather than advanced or competitive athletes–and addresses contexts in emerging markets such as China, where rapid growth has outpaced coach education and where instructional quality and safety literacy remain pressing concerns.

### 2.3 Enhancing coaching quality to increase learners intention to continue lessons

Therefore, in order to improve the effectiveness of snowboard coach training programs, and in light of the discussions in Section 2.1 about the integration of technical, pedagogical, and interpersonal competencies, as well as the focus of Section 2.2 on **loyalty**, this study focuses on how to strengthen these three core coaching competencies to stimulate the **loyalty** of learners to persist in lessons. Existing research suggests that systematic coaching training and support exert a significant impact on learner retention and subsequent lesson-taking behavior. For example, in a quasi-experimental study on academic tutoring programs, Alzen et al. (2021) found that the students who received coaching not only achieved higher academic performance, but also demonstrated stronger follow-up engagement. Furthermore, in the field of music training, Long and Lijia (2024) used a questionnaire-based investigation to examine how service quality, instructor expectations, and brand image affect learners intention to continue lessons and to recommend programs, revealing that effectively improving the perceived value of the learners' curriculum and overall satisfaction strengthens their desire to continue or recommend instruction. In sport settings, a large cross-sectional study of sports fitness centers (*n*= 606) found that higher perceived service quality—especially reliability, responsiveness, assurance, and empathy—raises satisfaction, which in turn increases trust and commitment and ultimately loyalty; these relationships are moderated by sport involvement (Huang and Kim, 2023). Building on these findings and taking into account the specific demands and characteristics of snowboard instruction, this study examines strategies to optimize technical guidance, refine teaching methods, and improve communication and motivational techniques within coach education. The aim is to introduce new perspectives on how to strengthen learners **loyalty (intention to continue lessons)** and referral intentions in snowboard lessons, thus contributing to a general increase in participation rates in winter sports.

## 3 Materials and methods

This study improved and optimized domestic snowboard coach training curricula according to Likert -type specifications Koo and Yang (2025); Hinkin et al. (1997). Based on the American Association of Snowboard **Instructors** (AASI)^2^ framework, key indicators for curriculum enhancements were established in three dimensions: interpersonal communication, technical proficiency, and pedagogical competence. Specific measurement elements were refined through in-depth interviews with senior domestic and international coaches. The scientific validity of the scale was verified using normality tests, hypothesis testing, and reliability—validity analyses. Finally, correlation analysis, global regression, and quantile regression methods were employed to investigate the impacts of the variables on **loyalty**, providing empirical evidence to optimize the coaching training curricula.

### 3.1 Item purification

**Figure 1:**
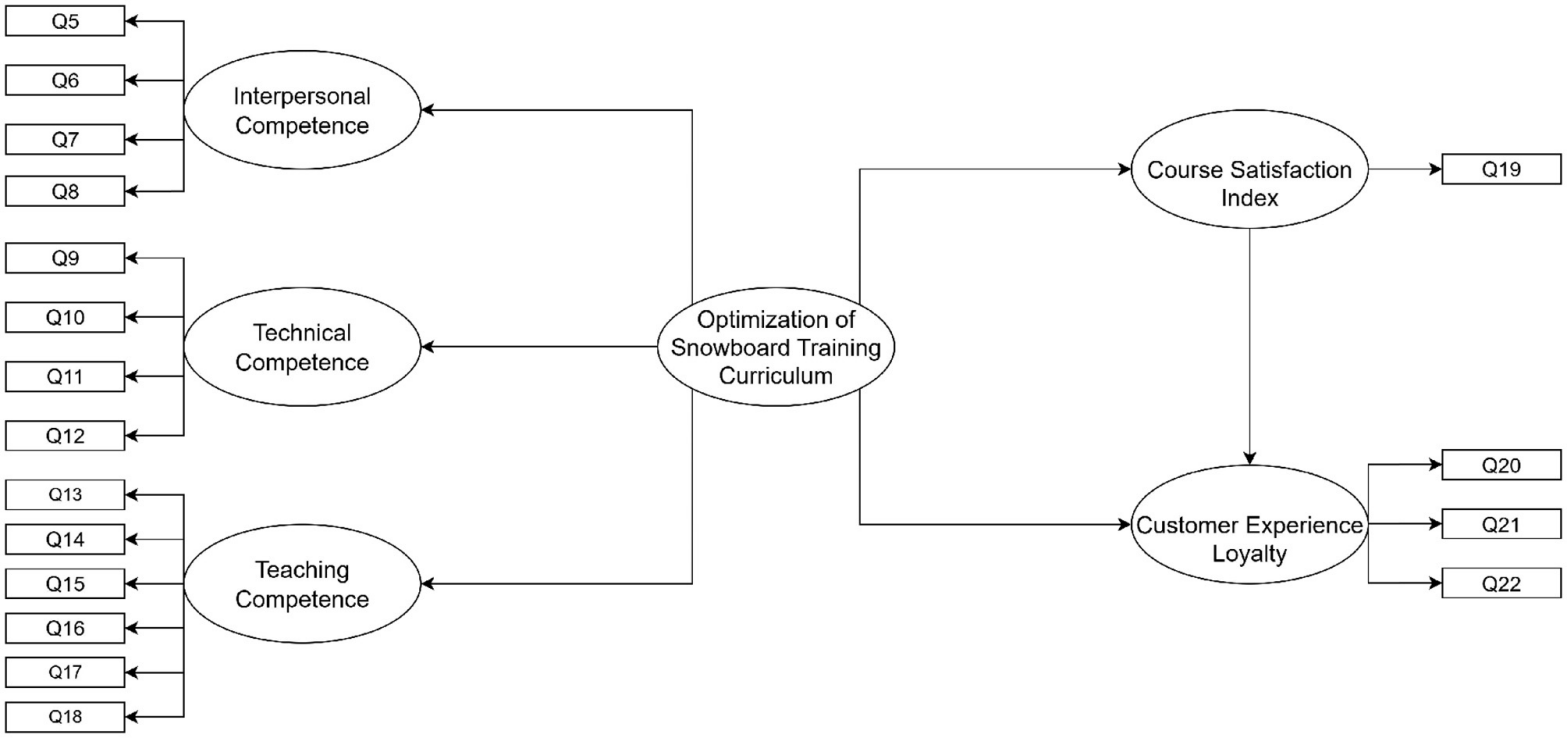
Snowboard_Instructor_Training_Augmentation-Optimization_Model (Loyalty is modeled as a three-item composite (Q20–Q22)). This framework is pre-factor rotation.

During the scale development phase (Stringfellow et al., 2024; Yin et al., 2025), this study conducted structured in-depth interviews with five internationally recognized snowboard coaches to define and refine the measurement indicators in each dimension. Specifically, the indicators for interpersonal and pedagogical competencies were based on the existing AASI **instructor** curriculum PSIA-AASI (2025) and the research of well-known scholars in the field, then further refined through practical insights from senior domestic coaches. Technical competency indicators were derived through comparative analysis between domestic and AASI training courses, identifying critical training components relevant to the Chinese **snowboarding** context. In addition, general satisfaction was included as a study outcome, with age, sex, snowboarding experience (< 1 season; 1–3 seasons; >3 seasons), and learning preferences Senior Specialist Manual (2008) (Visual, Auditory, Kinesthetic) serving as control variables. Detailed scale items are provided in the Appendix 1. The model is shown in Figure 1.

### 3.2 Study scope

This research selected Xinjiang's Koktokay Ski Resort and Hubei's Lvcongpo Ski Resort as study sites to ensure geographical and skill-level diversity, thus enhancing the comprehensiveness and representativeness of the findings.

**Koktokay ski resort** (46°47'N, 89°48'E), located in the Altai Mountains at 2,200–3,100 m above sea level, benefits from high-altitude topography and abundant natural snowfall, resulting in superior snow quality and a six-month operational season. Established in the early 20^th^ century and officially opened in 2020, it has become China's premier snowboard destination, featuring a highly skilled coaching team predominantly certified by international snowboard coach programs.

**Lvcongpo ski resort** (30°30'N, 110°20'E), located in the Wuling mountain range at an elevation of 1,500–1,800 m, is based on artificial snowmaking with shorter seasonal operations and a beginner-oriented infrastructure. Its coaching staff consists mainly of locally trained coaches with limited certification.

**Figure 2:**
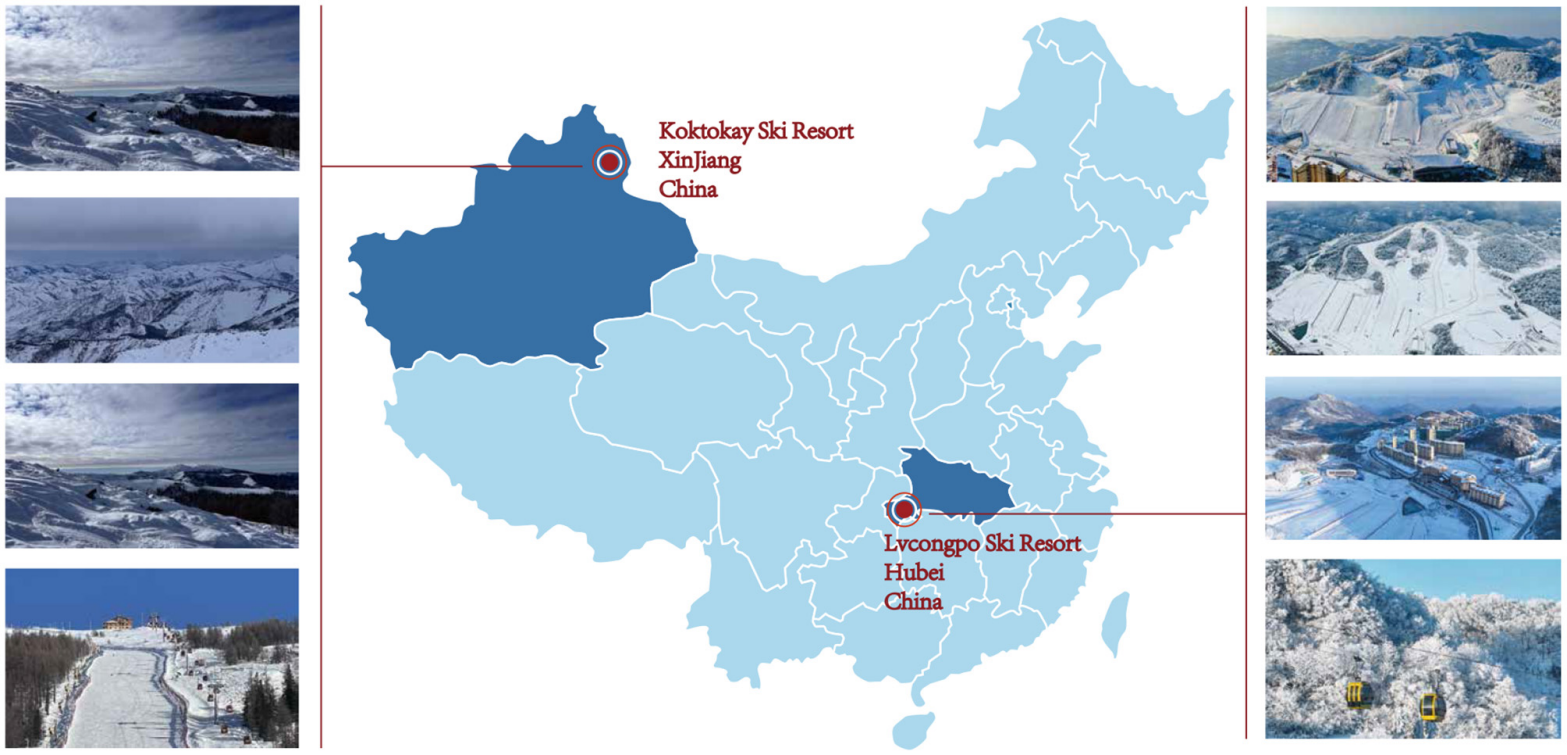
Study scope.

Table 1 shows the differences between the two ski resorts. By contrasting these northern and southern resorts in terms of natural conditions, historical development, and coach competency levels, this study provides a solid empirical foundation for analyzing the landscape of snowboard coaches in China. See Figure 2 for the exact location.

**Table 1:** Comparison of Keketohai and Lücongpo ski resorts.

**Criteria**	**Keketohai ski resort**	**Lücongpo ski resort**
Location	Altay Mountains, 2200–3100 m	Wuling Mountains, 1500–1800 m
History	Established in early 2000s, internationally renowned	Developed after 2020, emerging ski resort
Size	Large, diverse slopes for all levels	Small, limited slopes, suitable for beginners and families
Coaches	Highly skilled, internationally certified	Mostly local, less professional
Snow quality	Mainly natural, excellent, long season	Mostly artificial, short season
Typical clientele	Higher share of intermediate/advanced snowboarders, also many novices	High share of first-timers/beginners and families, also intermediates
Facilities	Well-equipped, integrated lodging, dining, entertainment	Basic, limited services

### 3.3 Data collection

This study was conducted during the early winter season (coinciding with the start of resort operations) using a stratified sampling method (Neyman, 1992; Spittle and Byrne, 2009). A representative sample of 251 snowboard participants were recruited across demographics of age. Of these, 250 valid respondents comprised 50.8% male and 49.2% female; ages ranged from 8 to 59 years (largest cohort at age 18, *n* = 32). Regarding snowboarding experience, 66.4% reported less than one season, 25.2% had 1–3 seasons, and 8.4% had more than three seasons. Preferred learning modalities (Senior Specialist Manual, 2008; Gilakjani et al., 2012; Ibrahim and Hussein, 2016) (multiple selections) were: kinesthetic 80.4%, visual 40.8%, and auditory 30.4%. All respondents were actively enrolled in snowboard lessons at the time of data collection and had been receiving coaching for a sufficient period to provide informed evaluations of the instruction. A mixed-methods approach that integrated the distribution of the online questionnaire and on-site fieldwork (including purposive on-site selection of snowboarders by age, sex, and observed skill level; brief post-lesson interviews on constraints in Chinese snow sports; and structured observations of coaching practice to identify common issues) ensured the completeness and reliability of the data. Systematic surveys were designed to capture regional variations in participants evaluations of instructional programs. All data were collected using the *WJX* platform.^3^ After collection, the data set was cleaned to remove incomplete or anomalous entries. The cleaned data were then exported in Excel format and subsequently imported into SPSS,^4^ where statistical analyses were performed (LIANG et al., 2023).

### 3.4 Measures and variables

#### 3.4.1 Primary outcome

*Loyalty* denotes a learners conative intention to maintain instruction with the same school/program. It is measured with three items (Q20–Q22 in Appendix 1): (i) re-enroll in lessons at this school; (ii) enroll in the next/advanced level; and (iii) recommend the course to friends or family. All items use a 5-point agreement scale (1 = Strongly disagree to 5 = Strongly agree). The composite loyalty index is the mean of Q20–Q22 (higher values indicate greater loyalty).

#### 3.4.2 Independent variables

Predictor constructs were based on three item pools–*Interpersonal Competence, Technical Competence*, and *Teaching Competence*—listed in Appendix 1. Composite predictors were computed from these items; their derivation and naming are presented subsequently (see Appendix 2).

#### 3.4.3 Additional covariates

*Overall satisfaction (OS)* (single 5-point evaluative item) and *snowboarding duration (SD)* (self-reported cumulative experience) were included as covariates.

### 3.5 Analytical procedures

#### 3.5.1 Measurement model and preliminary analysis

At this stage, the study performed normal distribution tests (DAgostino, 2017), hypothesis tests (Klein et al., 2003), and reliability and validity analyses (Roberts et al., 2006) to ensure both data suitability and measurement consistency. Normal distribution tests helped confirm whether the continuous variables met the assumptions required for parametric methods, guiding subsequent analytical choices. Hypothesis testing was used to determine whether control variables significantly influenced the dependent variable, thus maintaining analytical rigor. Meanwhile, reliability analysis assessed the internal consistency of the measurement scale and validity analysis—which includes factor analysis supported by the Kaiser–Meyer–Olkin (KMO)^AU^ measure of sampling adequacy and Bartletts test of sphericity (Tobias and Carlson, 1969)—verified that the measured indicators accurately captured the underlying constructs. For clarity, the KMO index evaluates sampling adequacy for factor analysis and ranges from 0 to 1; values ≥0.60 are typically considered acceptable, ≥0.80 meritorious, and ≥0.90 excellent. In addition, for the three-item loyalty scale (Q20–Q22), internal consistency was high (Cronbachs α = [insert value]), and exploratory factor analysis supported a single-factor solution (eigenvalue >1; all loadings > [insert threshold]); accordingly, a composite loyalty index (mean of Q20–Q22) was used in subsequent analyses.

#### 3.5.2 Correlation and regression analysis

**Outcome and screening** The dependent variable is the composite loyalty index (mean of Q20–Q22). Following preliminary checks, Spearmans rank correlation (Gogtay and Thatte, 2017) was used as a bivariate screen to gauge the strength and direction of associations among variables.

**Modeling strategy and justification** Two complementary estimators were employed. Ordinary least squares (OLS) summarizes the *conditional-mean* association between coaching competencies and loyalty; heteroskedasticity-robust standard errors are reported given the bounded Likert-type outcome (1–5). To characterize distributional heterogeneity and reduce sensitivity to outliers and skewness typical of Likert-type indices, quantile regression (QR) (Koenker and Hallock, 2001) estimates conditional effects at selected quantiles (τ∈{0.05, 0.25, 0.50, 0.75}). QR thus reveals whether the same competency has differential impacts for low-, mid-, and high-loyalty learners, providing segment-specific inference that complements the OLS average.

**Estimation details** The OLS specification Kiers (1997) included rotated dimensions, overall satisfaction (OS)^AU^, and snowboarding duration (SD)^AU^; multicollinearity was checked via variance inflation factors (all < 5). The same covariate set was estimated at each QR quantile to enable distributional comparisons.

## 4 Results

The research findings are organized into two stages: the first stage addresses the Measurement Model and Preliminary Analysis, while the second stage focuses on Correlation and Regression Analysis.

### 4.1 The first stage

#### 4.1.1 Significance analysis of control variables

This section performs a significance analysis of the control variables to determine whether sex, age, snowboarding duration, and learning preferences should be incorporated as control variables in subsequent regression modeling. Initially, normality tests (e.g., Shapiro–Wilk or Kolmogorov–Smirnov) are performed to assess the characteristics of the data distribution. Based on the results of the normality tests, parametric tests (e.g., *t* tests, ANOVA) are applied if the data follow a normal distribution, whereas nonparametric tests (e.g., Mann–Whitney U, Kruskal–Wallis) are selected for nonnormally distributed data. This methodological approach ensures the rigorous selection of variables for regression modeling.

##### 4.1.1.1 Normal distribution tests

The results of the Shapiro–Wilk normality test for all variables showed that the *p*-values were significantly less than 0.05, indicating that none of the variables followed a normal distribution. This finding necessitates the use of nonparametric statistical methods in subsequent analyses, such as the Mann–Whitney U test and the Kruskal–Wallis test.

##### 4.1.1.2 Hypothesis tests

For the binary variable gender, the Mann–Whitney U test was applied, while the Kruskal–Wallis H test was used for the multicategorical variables age, snowboarding duration, and learning preferences. The significance analysis revealed that gender (*p*>0.05), age (*p*>0.05), and learning preferences (*p*>0.05) did not show statistically significant differences at the predefined threshold, indicating that there was no significant variability between the groups in these variables. However, the duration of **snowboarding** showed a significant effect (*p* < 0.05) on the distribution of responses to the survey item “Are you satisfied with the coach's ‘observation–evaluation–guidance analytical process in the current snowboarding course?” (Item Q18), suggesting an association between **snowboarding duration** and variability in continuous outcome measures. Consequently, **snowboarding duration** was included as a control variable in subsequent regression analyses to account for its potential confounding effects on the dependent variable.

#### 4.1.2 Reliability, validity, and factor analysis

##### 4.1.2.1 Reliability analysis

The reliability analysis of the questionnaire for the development and optimization of snowboard coach training programs in China demonstrated an exceptionally high internal consistency, with a Cronbach's alpha coefficient of 0.984 (see Table 2).

**Table 2:** Reliability analysis results.

**Cronbach's alpha**	**Number of items**	**Sample size**
0.984	22	250

##### 4.1.2.2 Validity analyses

The validity analysis of the questionnaire for the development and optimization of snowboard coach training programs in China demonstrated excellent construct validity. The KMO sampling adequacy measure reached 0.970 and the significance of the Bartlett test showed significant results (*p* < 0.001), which confirms the high suitability of the data for factor analysis (see Table 3).

**Table 3:** Validity analysis results.

**KMO value**	**Bartlett test significance**	**Number of items**	**Sample size**
0.970	< 0.001^***^	22	250

^***^: *p* < 0.001; ^**^: *p* < 0.01; ^*^: *p* < 0.05, statistically significant correlation. KMO, Kaiser-Meyer-Olkin measure of sampling adequacy. Bartletts test of sphericity tests H_0_: the correlation matrix is an identity matrix; a significant result indicates the data are suitable for factor analysis.

##### 4.1.2.3 Factor analysis

Factor analysis was conducted using SPSS. The Kaiser–Meyer–Olkin (KMO) measure exceeded 0.6 and Bartlett's sphericity test showed significance (*p* < 0.05), confirming strong intervariate correlations and suitability for factor analysis. Principal Component Analysis (PCA) extracted three common factors with a cumulative variance contribution rate of 83.04%, demonstrating a rational three-dimensional structure (see Table 4). The rotated component matrix revealed all factor loadings >0.5, confirming robust validity. Based on the rotation matrix patterns, the three factors were labeled as: **(1) Teaching Interaction and Feedback (TIF); (2) Motion Guidance and Optimization (MGO); and (3) Effectiveness Assessment and Customization (EAC)** (see Appendix 2).

**Table 4:** Total variance explained.

**Component**	**Initial eigenvalues**			**Extraction sums of squared loadings**			**Rotation sums of squared loadings**		
	**Total**	**Variance %**	**Cumulative %**	**Total**	**Variance %**	**Cumulative %**	**Total**	**Variance %**	**Cumulative %**
1	10.738	76.701	76.701	10.738	76.701	76.701	4.328	30.914	30.914
2	0.516	3.686	80.388	0.516	3.686	80.388	4.081	29.152	60.066
3	0.371	2.653	83.040	0.371	2.653	83.040	3.216	22.974	83.040

Extraction method: principal component analysis.

### 4.2 The second stage

#### 4.2.1 Correlation analysis

Spearman's correlation analysis evaluated relationships between TIF, MGO, EAC, OS and SD^AU^. The results indicated non-significant associations for the duration of snowboarding (SD) (*p*>0.05), while the correlations between the remaining four variables reached statistical significance (*p* < 0.01) (see Table 5).

**Table 5:** Correlation Matrix.

**Variable**	**TIF**	**MGO**	**EAC**	**OS**	**Loyalty**	**SD**
TIF	1.000	0.223^***^	0.222^***^	0.628^***^	0.594^***^	0.067
MGO	0.223^***^	1.000	0.143^*^	0.539^***^	0.526^***^	0.082
EAC	0.222^***^	0.143^*^	1.000	0.552^***^	0.525^***^	0.089
OS	0.628^***^	0.539^***^	0.552^***^	1.000	0.806^***^	0.029
Loyalty	0.594^***^	0.526^***^	0.525^***^	0.806^***^	1.000	0.115
SD	0.067	0.082	0.089	0.029	0.115	1.000

^***^p < 0.001; ^**^p < 0.01; ^*^p < 0.05. TIF, Teaching Interaction and Feedback; MGO, Motion Guidance and Optimization; EAC, Effectiveness Assessment and Customization. OS, Overall Satisfaction; SD, **Snowboarding** Duration. Loyalty = mean of Q20–Q22 (higher scores indicate greater loyalty).

#### 4.2.2 Regression analysis

##### 4.2.2.1 A global ordinary least squares linear regression

**Figure 3:**
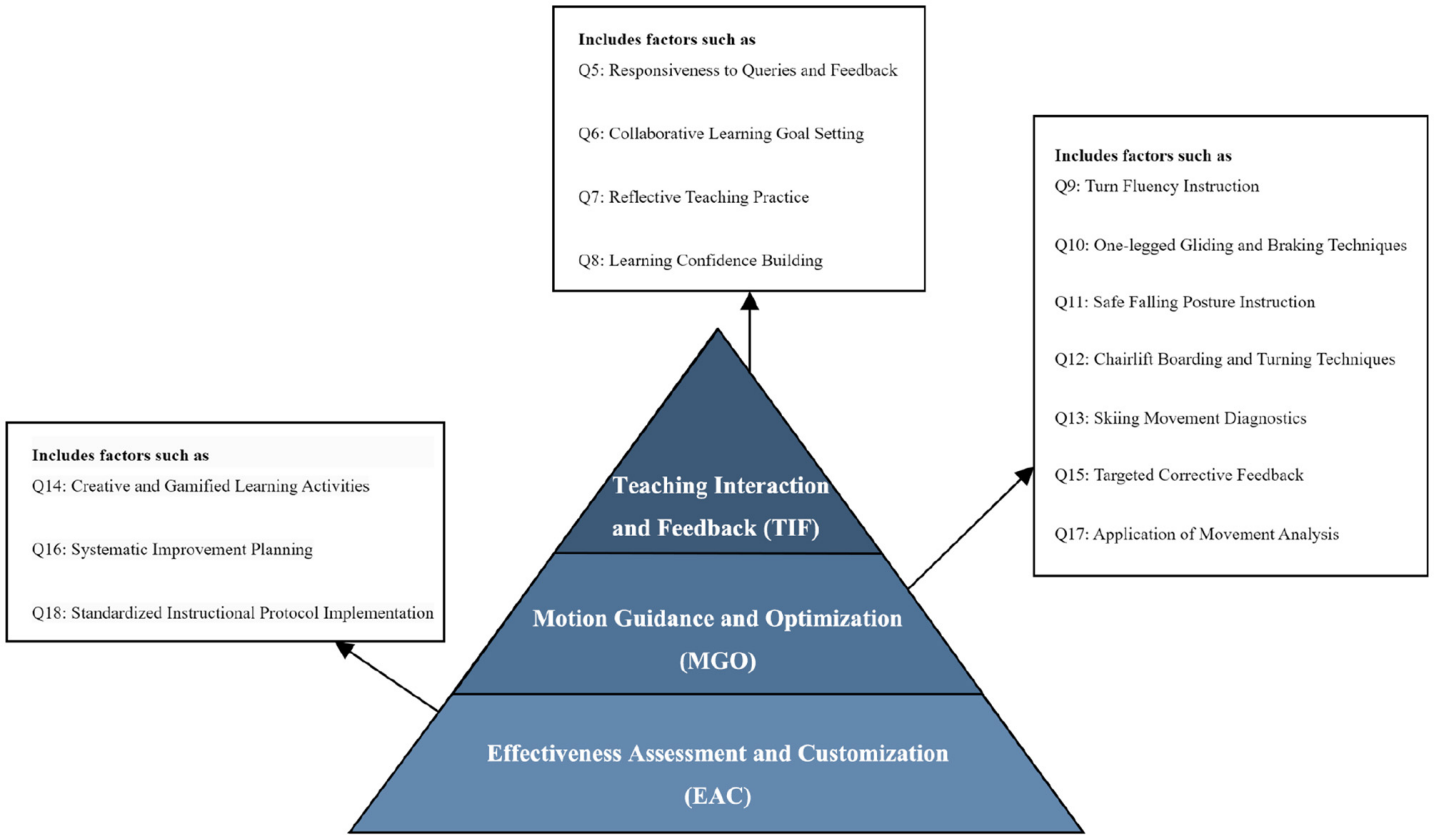
Competency pyramid based on OLS standardized coefficients. Darker shading indicates a larger standardized effect size (β) in the OLS model (see Table 6).

TIF, MGO, EAC, OS and SD were included in the regression model. Despite the non-significance of SD in the previous correlation analysis (*p*>0.05), it was retained based on theoretical relevance. The adjusted *R*^2^ of 0.754 indicated a strong fit to the model. The regression results revealed significant positive effects of TIF (*p* < 0.01), MGO (*p* < 0.01), EAC (*p* < 0.05) (see Figure 3) and OS (*p* < 0.05) on loyalty, while SD did not show a significant association (*p*>0.10). The variance inflation factors (VIFs) between 1 and 5 confirmed minimal multicollinearity, supporting the robustness of the model (see Table 6).

**Table 6:** Regression analysis results.

**Dependent variable**	**Independent variable**	**Unstandardized coefficients**		**Standardized coefficients**	**Sig**	**VIF**
		**B**	**Std err**	**Beta**		
**Loyalty**	Constant	3.697	0.288	–	< 0.001^***^	–
	SD	0.043	0.033	0.042	0.190	1.031
	TIF	0.305	0.033	0.463	< 0.010^**^	2.553
	MGO	0.272	0.029	0.413	< 0.010^**^	1.961
	EAC	0.232	0.029	0.351	< 0.010^**^	1.916
	OS	0.162	0.062	0.171	< 0.050^*^	4.375
**Adjusted** ***R***^**2**^						0.754

^***^ *p* < 0.001; ^**^ *p* < 0.01; ^*^ *p* < 0.05. Loyalty = mean of Q20–Q22 (higher scores indicate greater loyalty).

##### 4.2.2.2 Quantile regression

Using quantile regression, the analysis examined how these independent variables vary between different loyalty quantiles. The results are as follows (see Table 7 and Supplementary Figure 1):

(1) **Overall model fit**: The *R*^2^ values differ by quantile. In the 5% quantile, *R*^2^ = 0.4911, which increases to 0.6884 in the 25% quantile, indicating stronger explanatory power between loyalty levels 5% and 25%. In the 50% quantile, *R*^2^ = 0.6809, then decreases to 0.3811 in the 75% quantile, suggesting a weaker explanatory capacity between 50% and 75%.
(2) **Variable effects**:
  (a) TIF is significant (*p* < 0.05) across all quantiles, with coefficients rising from 0.2358 (5% quantile) to 0.3887 (50%), then slightly dropping to 0.3355 (75%). This pattern implies a consistently positive influence, especially pronounced at lower to moderate loyalty levels.
  (b) MGO is also significant (*p* < 0.05) in all quantiles, but its coefficient decreases to 0.1994 in the 75% quantile, suggesting a weaker effect among **learners** at higher loyalty levels.
  (c) EAC is significant (*p* = 0.000) in the 25%, 50%, and 75% quantiles, but not significant (*p* = 0.100) in the 5% quantile, indicating a stronger impact on medium- to high-loyalty **learners** compared to those with low loyalty.
  (d) SD is significant (*p* = 0.036) only in the 50% quantile, implying a minimal effect at moderate loyalty levels and no apparent effect at extremes.
  (e) OS shows marginal significance (*p* < 0.05) in the 5% quantile and strong significance (*p* < 0.001) in the 25% and 50% quantiles. Its largest unstandardized coefficient (0.517) emerges in the 5% quantile, indicating that despite the weaker significance, its relative impact on **learners** at the lowest level of loyalty is relatively high.

**Table 7:** Quantile regression results.

**Variable**	**5% Quantile**	**25% Quantile**	**50% Quantile**	**75% Quantile**
Constant	1.463	4.205^***^	4.074^***^	4.549^***^
(*P*-value)	(0.063)	(0.000)	(0.000)	(0.000)
TIF	0.236^*^	0.394^***^	0.389^***^	0.336^***^
(*P*-value)	(0.012)	(0.000)	(0.000)	(0.000)
MGO	0.253^**^	0.352^***^	0.323^***^	0.199^***^
(*P*-value)	(0.003)	(0.000)	(0.000)	(0.000)
EAC	0.146	0.295^***^	0.284^***^	0.241^***^
(*P*-value)	(0.100)	(0.000)	(0.000)	(0.000)
SD	0.093	−3.69E-08	1.146e-07^*^	5.894e-07
(*P*-value)	(0.083)	(1.000)	(0.036)	(1.000)
OS	0.517^**^	0.053^***^	0.084^***^	0.0131
(*P*-value)	(0.004)	(0.000)	(0.000)	(0.728)
Pseudo *R*^2^	0.491	0.688	0.681	0.381

^***^ *p* < 0.001; ^**^ *p* < 0.01; ^*^ *p* < 0.05. Loyalty = mean of Q20–Q22 (higher scores indicate greater loyalty).

## 5 Discussion

The discussion is anchored in three result blocks–measurement structure, OLS associations, and quantile heterogeneity—and then outlines how these results inform mechanisms and practice.

(1) **Three-dimensional competency structure**. Factor analysis refined the coach competency model (*Interpersonal Competence, Technical Competence*, and *Teaching Competence*) into three dimensions: *Teaching Interaction and Feedback (TIF)*^AU^, *Motion Guidance and Optimization (MGO)*, and *Effectiveness Assessment and Customization (EAC)*. This structure underpins subsequent interpretation.
(2) **OLS associations with loyalty**. After adjustment for selected controls, ***TIF***, ***MGO***, ***EAC***, and *overall satisfaction (OS)* show positive, statistically significant associations with *loyalty*, whereas *snowboarding duration (SD)* is not significant. Diagnostics indicate acceptable multicollinearity (all VIFs < 5) and good explanatory power (Adjusted *R*^2^ = 0.754).
(3) **Quantile heterogeneity across loyalty levels**. Effects are not uniform across the loyalty distribution. ***TIF1*** **and** ***MGO*** remain significant at all examined quantiles, with the largest marginal influence around the *25th percentile* (low-medium loyalty). ***EAC*** becomes comparatively more salient toward the *median-upper* quantiles. *OS* exhibits stronger leverage in the *low-medium* range. *SD* shows small and inconsistent signals (occasional significance around middle quantiles), suggesting limited robustness.

Building on these results, the discussion proceeds in four steps: (i) mapping the three competency dimensions to ***Self-Determination Theory*** **(SDT)** to clarify autonomy, competence, and relatedness pathways; (ii) synthesizing *OLS* and *quantile* evidence into implications for *coach-learner interactions* and *program design*; (iii) contextualized operational implications across resort settings, with an emphasis on *short-season and intermittently accessible environments*. This emphasis reflects both the national promotion priority for regions with less natural snow reliability and the present results showing larger marginal gains at lower loyalty quantiles; and (iv) critical reflection and transferability, situating the findings within broader outdoor sports literature and cross-cultural settings, and outlining avenues for future research.

### 5.1 An SDT reading of the three-factor competency model

Factor analysis yielded a three-factor structure—***TIF***, ***MGO1***, **and** ***EAC***—that is statistically sound (cumulative variance explained: 83.04%) and conceptually clearer than the traditional “interpersonal-technical-pedagogical” split. In plain terms, **TIF** captures interactional communication tied to goals and timely feedback; **MGO** links technical know-how with problem solving so that technique becomes learnable; and **EAC** centers on evaluation and personalization independent of motion-analysis content.

Within Self-Determination Theory^AU^, the initial three-way split would map neatly onto basic needs: interpersonal skills to *autonomy* and *relatedness*, technical skills to *competence*, and pedagogy spanning all three (Reeve and Cheon, 2021a; Ryan and Deci, 2000; Teixeira et al., 2012). The rotated solution suggests a different picture. **TIF** indicates that autonomy support and a sense of relatedness often co-occur within the same interaction (Deng et al., 2019). **MGO** shows that competence support arises not from “technique” alone but from how technique is decomposed, cued, and sequenced (Guadagnoli and Lee, 2004). **EAC** isolates an assessment-personalization axis, where competence information and recognition of individual goals appear side by side (Hattie and Timperley, 2007). In short, factors seem organized not only by content but also by how learners *experience* behaviors in terms of need support (informational vs. controlling) (Deci et al., 1999; Kluger and DeNisi, 1996). This also implies that elements traditionally grouped under “pedagogy” are distributed across **MGO** (rendering technique learnable) and **EAC** (assessment-personalization), while **TIF** captures the interpersonal core (Jang et al., 2010; Black and Wiliam, 1998; Nicol and Macfarlane-Dick, 2006).

Three theoretical takeaways follow. *First*, need supports are naturally coupled: a single instructional move–such as explaining the “why” behind a task–can meet more than one need at once, arguing against treating autonomy, competence, and relatedness supports as fully separate channels (Ryan and Deci, 2000; Reeve and Cheon, 2021a). *Second*, interaction climate shapes how other behaviors are interpreted: the same cue or assessment is likely to be internalized as competence support when embedded in an autonomy- and respect-supportive exchange, but may be read as controlling otherwise; analytically, this points to interaction effects between **TIF** and **MGO**/**EAC** (Jang et al., 2010; Sierens et al., 2009). *Third*, a *hypothesized* temporal order exists: **TIF** sets a supportive climate, **MGO** enables progress, and **EAC** consolidates understanding and personal fit–suggesting models that treat need support as an episode unfolding across these phases (Hattie and Timperley, 2007; Rosenshine, 2012).

Two caveats apply. Some coupling may reflect measurement features (item wording proximity, common-method variance); checks with alternative parcels or multi-method designs are warranted. In addition, the resort-based, face-to-face instructional context in China may strengthen the joint appearance of autonomy and relatedness in feedback, cautioning against assumptions of universality without cross-cultural replication.

Overall, the rotated three-factor solution reframes coach competencies as interdependent need-support constructs rather than three separate content silos, sharpening **SDTs** application by focusing on learners experience of behaviors (informational vs. controlling) and motivating interaction and sequencing hypotheses for future testing.

### 5.2 Integrating OLS and quantile evidence: implications for coach–learner interactions and program design

The OLS model indicates that ***TIF***, ***MGO***, ***EAC***, **and** ***OS*** are positively and significantly associated with *loyalty*, whereas *SD* is not significant; diagnostics suggest acceptable multicollinearity (all VIFs < 5) and substantial explanatory power (Adjusted *R*^2^ = 0.754).

Quantile estimates reveal heterogeneous leverage across the loyalty distribution. ***TIF*** **and** ***MGO*** remain significant throughout, with the strongest marginal influence around the *25th percentile*. ***EAC*** is non-significant at the *5th percentile* but becomes significant and broadly stable from the *25th* through the *75th percentiles*, with a slight tapering at the upper end. ***OS*** shows a pronounced spike at the *5th percentile*, drops sharply by the *25th percentile*, remains detectable but small around the *50th percentile*, and is negligible at the *75th percentile*. ***SD*** exhibits minimal and inconsistent effects. Consistent with the Self-Determination Theory^AU^ reading in Section 5.1, this pattern aligns with a need-deficit logic: stronger **TIF/MGO** effects at lower quantiles indicate larger returns when autonomy and competence are initially unmet (Reeve and Cheon, 2021a; Vansteenkiste and Ryan, 2013); the broadly stable yet slightly tapering **EAC** from the 25th-75th percentiles reflects competence consolidation with diminishing marginal gains; and the **OS** spike at the 5th percentile suggests contextual satisfiers are most consequential before self-endorsed motivation stabilizes (Faullant et al., 2008).

Taken together, these results suggest three design principles. *Functional complementarity* (Adams et al., 2017): **TIF, MGO, and EAC** act as an integrated system rather than substitutes–interactional climate **(TIF)** enables guidance and assessment to be received as informational, **MGO** provides skill clarity, and **EAC** completes the learning loop through progress information and personalization, consistent with Self-Determination Theory^AU^ on relatedness, competence, and autonomy as interdependent needs. *Deficit-sensitive responsiveness* (Vansteenkiste and Ryan, 2013): larger marginal effects at lower quantiles indicate greater returns where basic psychological needs are less satisfied, implying higher intensity of need-supportive behaviors for at-risk segments. *Developmental contingency* (Jang et al., 2016): the prominence of specific competencies shifts with progression–**TIF/MGO** dominate earlier phases when skills and confidence are fragile; **EAC** becomes steadily relevant from the 25th percentile onward but tapers at the very top; **OS** is most consequential at the extreme low end and quickly attenuates thereafter.

For coach–learner interactions, a segmentation-aware emphasis follows without prescribing rigid tiers. At lower loyalty levels (5th-25th percentiles), clear goal alignment, concise external-focus cueing, short success cycles, and friction reduction are pivotal, while **EAC** remains light-touch and informational to avoid controlling interpretations (Hattie and Timperley, 2007); **OS** is especially consequential at the very low end (Tuu and Olsen, 2010). Around the median (50th percentile), **TIF/MGO** remain central but benefit from more structured EAC–transparent criteria, individualized checkpoints, and visible progress markers–to prevent plateau (Shute, 2008). At upper loyalty levels (75th percentile and above), **EAC** increasingly carries personalization and mastery-oriented goals; **TIF** sustains autonomy and respect, and **MGO** shifts toward fine-tuning, with OS effects typically negligible.

At the program level, a compact session script aligns with the evidence without imposing fixed proportions: an opening that establishes rationale and shared objectives (**TIF**-emphasized), a development phase of progressive tasks with bandwidth-appropriate feedback and external-focus cueing (**MGO**-emphasized), and a consolidation phase that provides informational assessment and next-step personalization (**EAC**-emphasized). This sequencing mirrors the observed shifts in leverage across quantiles and offers a coherent bridge from statistical results to interactional routines and curricular structure, and is consistent with the *temporal hypothesis* advanced in Section 5.1.

Future work could examine generalizability to other individual sports and test the temporal stability of these loyalty-competency relations in longitudinal designs; identifying reliable indicators for assessing current loyalty levels would further support differentiated application.

### 5.3 Contextualized operational implications across resort settings

Translating the evidence into operations calls for sensitivity to setting-specific constraints. The sampling frame spans both northern and southern resorts; however, many target markets for nationwide participation growth operate under *short seasons* and *intermittent access*, conditions more common at lower latitudes (An et al., 2019). Emphasizing this context reflects both a national promotion priority where natural-snow reliability is lower (An et al., 2019) and the quantile results in Section 5.2 showing larger marginal gains at lower loyalty quantiles.

At a general level, three levers follow directly from the combined **SDT** reading in Section 5.1 and the OLS–quantile pattern. First, organize delivery around the *complementary bundle* of **TIF** (interactional climate) (Reeve and Cheon, 2021a), **MGO** (skill clarity) (Guadagnoli and Lee, 2004), and **EAC** (informational assessment/personalization) (Hattie and Timperley, 2007), rather than treating competencies as substitutes. Second, *prioritize need-deficit segments*: the lower end of the loyalty distribution responds most to autonomy-, relatedness-, and competence-supportive inputs, so entry points, first lessons, and early return visits warrant disproportionate attention (Tuu and Olsen, 2010; Professional Ski Instructors of America, PSIA). Third, adopt *sequenced sessions* that align with the hypothesized temporal order–open with climate-setting (**TIF**), progress via tightly scoped tasks (**MGO**), and close with informational feedback and next-step personalization (**EAC**)–while keeping proportions flexible across programs (Rosenshine, 2012).

Under short-season and intermittently accessible conditions, several adaptations help preserve instructional quality despite compressed practice windows, artificial/firm surfaces, and rotating staff (Ismert and Petrick, 2004; Collins and Collins, 2016; Almqvist et al., 2022). For **TIF**, standardize brief onboarding scripts at lifting or staging nodes to align expectations and co-define a single session goal (Professional Ski Instructors of America, PSIA); use queue-side micro-feedback (“one cue per pass”) to maintain flow during peaks (Salmoni et al., 1984; Wulf, 2013); stabilize messages across seasonal staff with concise multilingual cue cards and simple expectation boards (PSIA–AASI Western Division, 2018); adopt handoff protocols so that when instructors rotate, subsequent sessions reopen with prior goals and the last effective cue (Müller et al., 2018). For **MGO**, favor friction-management progressions that match firm/artificial snow (e.g., flatland edging and pressure drills → low-angle traverses → J/garland patterns → linked turns) (American Association of Snowboard Instructors (AASI), 2022), use terrain-based learning features and low-consequence corridors for early wins (Almqvist et al., 2022; Snow Operating, LLC, 2015), and include quick stance/equipment checks at session start to mitigate fit issues amplified by hard surfaces; when thaw or crowding constrains repetitions (American Association of Snowboard Instructors (AASI), 2013), substitute short indoor/video briefings to retain instructional density (Mödinger et al., 2022). For **EAC**, rely on minimal but visible progress indicators (two observable markers per session) (Hattie and Timperley, 2007), simple “learner passport” records that survive staff turnover, and short-cycle (about one to two weeks) personalization plans aligned to holiday peaks (Falk and Hagsten, 2016); where consent allows, anchor the next visit with a brief reference clip and a QR-linked next-step card (van der Meer et al., 2024). These adaptations aim to keep feedback informational, protect early competence gains, and maintain personalization despite staffing churn.

Two cross-cutting points follow from the quantile evidence. Overall satisfaction (OS)^AU^ shows a pronounced spike at the 5th percentile and attenuates quickly thereafter (Faullant et al., 2008; Tuu and Olsen, 2010), indicating that onboarding frictions, clarity of procedures, perceived safety, and service reliability are disproportionately consequential for very low-loyalty segments (Miragaia et al., 2016; Kyle et al., 2010; Hasan et al., 2017); practical emphasis should therefore fall on removing early barriers rather than adding amenities (Berry et al., 2002). **EAC** is broadly stable from the 25th to 75th percentiles with slight tapering at the upper end, consistent with competence consolidation once basic interactional and guidance needs are met (Guadagnoli and Lee, 2004); informational, self- or criterion-referenced feedback is preferable to rank-centric grading to avoid controlling interpretations in these ranges (Butler and Nisan, 1986).

Implementation and monitoring should remain light but consistent (Glasgow and Riley, 2013). Segmentation can be operationalized with simple rules (e.g., proxy indicators such as visit count or completion of foundational tasks) to adjust emphasis among **TIF**/**MGO**/**EAC** without rigid tiers (Fader et al., 2005). Suggested KPIs include repeat-enrollment rates, post-session rebooking within defined windows, progression through observable technique milestones, and incident-free sessions during early lessons (Dick and Basu, 1994). Finally, transferability is conditional: these recommendations are designed for short-season/limited-access contexts and should be calibrated for resorts with longer seasons, deeper snowpacks, or different participant mixes (Steiger et al., 2019), as discussed in the subsequent reflection on limitations and cross-setting applicability.

### 5.4 Critical reflection and transferability

Situated within broader debates in outdoor and lifestyle sports, the present synthesis supports a view of coaching as *need-supportive* practice enacted through complementary instructional levers Curran and Standage (2017). The three-factor structure—TIF, MGO and EAC^AU^—aligns with models that foreground interactional climate, skill clarity, and informational feedback. Beyond confirming this alignment, the distributional evidence locates where each lever matters most along the loyalty spectrum, thereby refining how *need support* is understood in settings marked by environmental volatility, intermittent access, and diverse participant profiles Rios-Avila and Maroto (2024).

A first reflection concerns *theoretical triangulation*. The **TIF–MGO–EAC** bundle is consistent with SDT^AU^ but also sits comfortably alongside complementary perspectives. From an *ecological dynamics* or constraints-led lens, **MGO** can be read as shaping task and environmental constraints so that functional movement solutions become attuned and discoverable (Renshaw and Chow, 2019); **TIF** modulates the interpersonal context that regulates arousal and exploration (Reeve and Cheon, 2021a); and **EAC** provides informational coupling through criterion- or self-referenced signals that stabilize learning (Panadero, 2023). Expectancy–value viewpoints similarly illuminate why clarity and early progress **(MGO/EAC)** elevate perceived competence and task value, while **TIF** frames social meaning and belonging. The convergence of these accounts suggests that the observed “bundle advantage” is not merely additive but reflects how interpersonal climate, task design, and feedback co-determine experiential valence (informational vs. controlling) and, in turn, internalization (Reeve and Cheon, 2021a).

A second reflection addresses *transfer across activity types* (Pan, 2020). In individual outdoor sports that are episodic, risk-mediated, or novelty-rich (e.g., climbing, surfing, mountain biking, skateboarding), the same bundle logic is likely to generalize (Eastabrook and Collins, 2021): autonomy- and relatedness-supportive interaction **(TIF)** scaffolds entry; tightly scoped progressions and external-focus cueing **(MGO)** translate uncertainty into learnable steps; informational assessment and personalization **(EAC)** consolidate progress. Where access is more continuous and repetition plentiful (e.g., long-season alpine regions or high-reliability indoor facilities), relative leverage may shift toward richer **EAC** pathways and finer-grained **MGO**, with **TIF** maintaining climate rather than driving early retention Collins and Collins (2015). Team sport contexts may also benefit, but additional meso-level dynamics enter (peer climate, role clarity, shared goals), implying that **TIF** must be extended to group-level relatedness and autonomy structures, and that **EAC** should incorporate collective as well as individual indicators (Collins et al., 2016).

A third reflection considers *cross-cultural transfer* (Thomas and Kobayashi, 2014). Need-supportive coaching does not look the same across cultures. In higher power-distance or high-context settings, “autonomy support” is best expressed as *choice within structure*: the coach first states a clear goal and safety limits, offers two or three viable next steps, and briefly explains the rationale for each, then invites the learner to choose (Chirkov et al., 2003). This preserves agency without sacrificing clarity or face. “Relatedness” is often built through group rituals and shared milestones rather than dyadic exchange alone. For ***MGO*** **and** ***EAC***, direct cues can remain informational when the reason is explicit and delivered respectfully; progress feedback is better framed around mastery and self-referenced growth than rank comparisons to avoid a controlling tone. These adaptations align with **SDTs** focus on the *experienced* quality of behavior: culture tends to shape the form of need support more than its function (Chen et al., 2015).

A fourth reflection concerns *resource and infrastructure gradients* (Deng et al., 2019; Wicker et al., 2013). The bundle is notably *low-tech viable*: **TIF** relies on communication quality (Wisniewski et al., 2020); **MGO** on principled task design (Tomlinson and McTighe, 2006); **EAC** on clear criteria and simple visibility of progress (Arnold, 2011). This makes the approach adaptable to programs with constrained equipment, staffing churn, or firm/artificial surfaces. In resource-rich environments, technology (e.g., video feedback, inertial sensors) can amplify **EAC** and **MGO** without displacing **TIF** (Mödinger et al., 2022), provided that data use remains informational and learner-centered. Where bandwidth or staffing is limited, lightweight artifacts (cue cards, simple progress trackers) can preserve personalization across instructors and visits (Salmoni et al., 1984; Wulf, 2013). Thus, transferability hinges less on equipment level than on maintaining informational tone and coherent sequencing across the bundle.

Finally, *equity and access* considerations broaden the interpretive frame (Thibaut et al., 2017). The pronounced sensitivity of entry segments to contextual satisfiers underscores how non-instructional frictions (clarity of procedures, perceived safety, wait times, affordability) interact with pedagogical levers to shape early loyalty (Miragaia et al., 2016; Kyle et al., 2010). Similar patterns are plausible in other fee-based, travel-dependent activities where participation costs are front-loaded and skills initially fragile (Thibaut et al., 2017; Berry et al., 2002). Embedding the **TIF–MGO–EAC** bundle within equitable service design—minimizing early barriers, clarifying pathways, and recognizing diverse starting points—is therefore central to transfer, particularly in regions or programs seeking to widen participation (Berry et al., 2002).

In sum, the evidence refines a need-support account of resort-based instruction by identifying a complementary competency bundle and by indicating where its leverage is strongest along the loyalty distribution. Transferability is promising across individual outdoor sports and varied facility ecologies, provided that cultural expression, access patterns, and resource conditions are respected. The central requirement is not a fixed protocol but fidelity to the *informational* character of interaction, guidance, and assessment, and to their coherent sequencing over the learners experience (Wisniewski et al., 2020; Chambers et al., 2013).

## 6 Limitation

Despite providing valuable information, this study faces several limitations that warrant attention:

(1) **Sample Coverage and Regional Disparities**: Drawing on two resorts, Keketohai in Xinjiang and Lücongpo in Hubei, this research explores the overall teaching quality of **snowboard** instructors in different tiers of Chinese **snowboard** resorts. However, the geographically limited sample excludes traditional winter sports regions in Northeast China (e.g., Yabuli in Heilongjiang, Beidahu in Jilin), as well as alpine resorts in low-latitude areas in the southern and southwestern parts of China. These regions may differ markedly in operational models, coach training systems, and user needs, potentially affecting the universality and representativeness of the findings.
(2) **Insufficient Coverage of Advanced User Needs in the Curriculum**: Although this study focuses on popular **snowboard** instruction scenarios, emphasizing basic skills such as single-foot gliding, chairlift use, and basic pressure application, it does not fully address the demands of advanced users. As **snowboarding** gains deeper traction in China, higher-level skills, such as carving techniques and complex terrain management, are becoming increasingly important. However, the current curriculum lacks a systematic advanced module and questionnaire items are heavily oriented toward basic skill satisfaction, overlooking critical dimensions (e.g., efficiency of movements, control on steep slopes) that matter to more experienced **snowboard learners**. Quantile regression shows limited explanatory power in the 75% quantile (*R*^2^ = 0.381), suggesting that the specific needs of advanced learners were not adequately captured.
(3) **Subjectivity in Teaching Ability Assessment**: This research relies on subjective learning satisfaction ratings (e.g., ‘learning content satisfaction) to evaluate course optimization and coaching proficiency, lacking objective technical indicators (such as **snowboard** performance metrics or completion rates). For example, high satisfaction with “motion guidance and optimization” could stem more from the communication style of the coach than from actual technical effectiveness. Moreover, the absence of video-based movement analysis tools prevents identifying subtle teaching flaws (e.g., incorrectly standardized demonstrations).

In summary, future studies should expand sample coverage to include traditional winter sports regions in northeast China and low-latitude alpine resorts in southern areas, exploring differences in operational modes and coaching systems to enhance generalizability and representativeness of the findings. Furthermore, introducing advanced modules targeting higher-level **snowboard learners** and establishing a multidimensional assessment system, spanning beginner to expert skills, would improve the relevance and impact of the curriculum. Objective data collection via pressure-sensitive snowboard equipment or real-time tracking of **snowboard learner** parameters (e.g., edging angles, center-of-mass shifts, turn symmetry) can facilitate the development of robust performance benchmarks. Leveraging computer vision to analyze coaching videos at a frame-by-frame level, benchmarking them against international standards (e.g., AASI manuals) can further generate detailed diagnostic reports on teaching efficacy. Together, these refinements would enable **more precise** and intelligent evaluations of instructor performance and learner development.

## 7 Conclusion

This study developed an enhanced snowboard coach training curriculum focused on three interrelated competencies–Teaching Interaction and Feedback (TIF), Motion Guidance and Optimization (MGO), and Effectiveness Assessment and Customization (EAC). Findings indicate that strengthening these competencies leads to more positive learning experiences and greater loyalty among learners, underscoring that effective coaching depends not only on technical expertise but also on the capacity to teach effectively and adapt to learner needs.

A key outcome is the recognition that snowboard learners benefit from stage-appropriate instructional approaches. Beginner, intermediate, and advanced recreational learners respond differently to coaching strategies, highlighting the need to tailor pedagogy to each learners stage of development. Adopting a stratified coaching model–emphasizing enjoyment and basic skills for novices, technical refinement for intermediate learners, and personal goal setting for advanced learners–can improve engagement and skill acquisition across levels. The results further indicate that such tailoring within coach education elevates program quality and promotes sustained lesson participation (loyalty).

The implications of this work extend to multiple domains:

(1) **Theoretical integration (SDT)**^AU^: *Framed by Self-Determination Theory (SDT)*, the competency bundle functions as complementary need support: TIF establishes an autonomy- and relatedness-supportive climate; MGO clarifies action and calibrates challenge to build competence; and EAC provides informational, criterion- or self-referenced feedback that consolidates competence while preserving autonomy through personalization. The *quantile pattern* (stronger effects at lower loyalty quantiles) is consistent with a need-deficit logic, and the implied sequencing (TIF → MGO → EAC) helps explain why complementary delivery strengthens internalization and, ultimately, loyalty.null
(2) **Coaching practice**: Evidence indicates that investment in interactive communication, individualized feedback, and ongoing assessment translates into better on-slope outcomes, fostering inclusive and motivating learning environments and improving newcomer loyalty.
(3) **Coach education**: The findings underscore the value of embedding pedagogical skill development in training and certification–ensuring that new instructors learn not only *what* to teach, but also *how* to teach effectively.
(4) **Sport pedagogy**: The study illustrates how pedagogical models can be operationalized in non-traditional physical activity settings; aligning instructional strategies with learner needs in active-leisure contexts helps bridge educational theory and coaching practice.

In sum, optimizing snowboard coach training through a competency-based model offers a pathway to elevate instructional quality, enhance learner development, and support sustained lesson participation and broader winter sport engagement, with SDT clarifying *why* the model works and *where* its leverage is greatest along the learner loyalty distribution.

## Data Availability

The raw data supporting the conclusions of this article will be made available by the authors, without undue reservation.

## Footnotes

^1^Southern low-latitude alpine ski resorts operate during a brief 2.5-month season (mid-December to early February) and rely on “migratory coaches” from northern China due to lack of local ski industry. These temporary instructors typically possess moderate technical skills and return north after the season, preventing continuous, systematic instruction for local learners.

^2^The Professional **Ski** Instructors of America and American Association of Snowboard Instructors (PSIA–AASI) is the world's largest non-profit organization for **ski and snowboard** education. It includes over 300 schools and 32,500 instructors, and is headquartered in Lakewood, Colorado, USA.

^3^Wenjuanxing is an online survey platform that provides tools for designing, distributing, and analyzing questionnaires. More details can be found at: https://www.wjx.cn/.

^4^SPSS (Statistical Package for the Social Sciences) is a statistical analysis software widely used in social sciences, market research, and business analytics. More details are available at https://www.ibm.com/products/spss-statistics.

## References

[B1] Adams N. Little T. D. Ryan R. M. (2017). “Self-determination theory,” in Development of Self-Determination Through the Life-Course (Cham: Springer), 47–54.

[B2] Allen J. B. Shaw S. (2009). “Everyone rolls up their sleeves and mucks in”: exploring volunteers motivation and experiences of the motivational climate of a sporting event. Sport Managem. Rev. 12, 79–90. 10.1016/j.smr.2008.12.002

[B3] Almqvist A. Pellegrini B. Lintzén N. Emami N. Holmberg H. Larsson R. (2022). A scientific perspective on reducing ski-snow friction to improve performance in olympic cross-country skiing, the biathlon and nordic combined. Front. Sports Active Living 4:844883. 10.3389/fspor.2022.844883 35392593 PMC8980609

[B4] Alzen J. L. Burkhardt A. Diaz-Bilello E. Elder E. Sepulveda A. Blankenheim A.. (2021). Academic coaching and its relationship to student performance, retention, and credit completion. Innovat. Higher Educ. 46, 539–563. 10.1007/s10755-021-09554-w

[B5] American Association of Snowboard Instructors (AASI) (2013). Adaptive Snowboard Guide. Lakewood, CO: PSIA-AASI.

[B6] American Association of Snowboard Instructors (AASI) (2022). “Snowboarding technical skills performance guide,” in Performance Guide. Lakewood, CO: PSIA-AASI.

[B7] An H. Xiao C. Ding M. (2019). The spatial pattern of ski areas and its driving factors in China: a strategy for healthy development of the ski industry. Sustainability 11:3138. 10.3390/su11113138

[B8] Arnold I. (2011). John Hattie: visible learning: a synthesis of over 800 meta-analyses relating to achievement. Int. Rev. Educ. 57, 219–221. 10.1007/s11159-011-9198-8

[B9] Behnam M. Sato M. Baker B. J. (2021). The role of consumer engagement in behavioral loyalty through value co-creation in fitness clubs. Sport Managem. Rev. 24, 567–593. 10.1080/14413523.2021.1880772

[B10] Behzadnia B. (2021a). The relations between students causality orientations and teachers interpersonal behaviors with students basic need satisfaction and frustration, intention to physical activity, and well-being. Phys. Educ. Sport Pedag. 26, 613–632. 10.1080/17408989.2020.1849085

[B11] Behzadnia B. Adachi P. J. Deci E. L. Mohammadzadeh H. (2018). Associations between students' perceptions of physical education teachers' interpersonal styles and students' wellness, knowledge, performance, and intentions to persist at physical activity: A self-determination theory approach. Psychol. Sport Exerc. 39, 10–19. 10.1016/j.psychsport.2018.07.003

[B12] Bergström M. Rosvold M. Sæther S. A. (2023). “I hardly have a problem […] I have my period quite rarely too”: female football players and their coaches perceptions of barriers to communication on menstrual cycle. Front. Sports Active Living 5:1127207. 10.3389/fspor.2023.1127207 37033882 PMC10076858

[B13] Berry L. L. Seiders K. Grewal D. (2002). Understanding service convenience. J. Market. 66, 1–17. 10.1509/jmkg.66.3.1.1850511670861

[B14] Black P. Wiliam D. (1998). Assessment and classroom learning. Assessm. Educ.: Principles, Policy & *Pract*. 5, 7–74. 10.1080/0969595980050102

[B15] Butler R. Nisan M. (1986). Effects of no feedback, task-related comments, and grades on intrinsic motivation and performance. J. Educ. Psychol. 78:210. 10.1037/0022-0663.78.3.210

[B16] Chambers D. A. Glasgow R. E. Stange K. C. (2013). The dynamic sustainability framework: addressing the paradox of sustainment amid ongoing change. Implementat. Sci. 8:117. 10.1186/1748-5908-8-117 24088228 PMC3852739

[B17] Chaves-Castro K. Morán-Gámez G. Nuviala A. Fernández-Martínez A. (2025). Green practices and sustainability as precursors to the intention to participate again in sporting events held in nature. Front. Sports Active Living 7:1541485. 10.3389/fspor.2025.1541485 40115439 PMC11922924

[B18] Chen B. Vansteenkiste M. Beyers W. Boone L. Deci E. L. Van der Kaap-Deeder J.. (2015). Basic psychological need satisfaction, need frustration, and need strength across four cultures. Motiv. Emot. 39, 216–236. 10.1007/s11031-014-9450-1

[B19] China National Vocational Qualification Training Program for Social Sports Instructors (2023). National Vocational Qualification Training Textbook for Social Sports Instructors: Skiing. Beijing: Higher Education Press of China.

[B20] China News Service (2025). Seeking a Guide at the Ski Resort but Ended up With an Unlicensed Instructor. Beijing: China News Service.

[B21] Chirkov V. Ryan R. M. Kim Y. Kaplan U. (2003). Differentiating autonomy from individualism and independence: a self-determination theory perspective on internalization of cultural orientations and well-being. J. Pers. Soc. Psychol. 84:97. 10.1037/0022-3514.84.1.97 12518973

[B22] Collins D. Collins L. Carson H. J. (2016). “If it feels right, do it”: Intuitive decision making in a sample of high-level sport coaches. Front. Psychol. 7:504. 10.3389/fpsyg.2016.00504 27148116 PMC4830814

[B23] Collins L. Brymer E. (2020). Understanding nature sports: A participant centred perspective and its implications for the design and facilitating of learning and performance. Annals Leisure Res. 23, 110–125. 10.1080/11745398.2018.1525302

[B24] Collins L. Collins D. (2015). Integration of professional judgement and decision-making in high-level adventure sports coaching practice. J. Sports Sci. 33, 622–633. 10.1080/02640414.2014.953980 25397633

[B25] Collins L. Collins D. (2016). Professional judgement and decision-making in adventure sports coaching: The role of interaction. J. Sports Sci. 34, 1231–1239. 10.1080/02640414.2015.1105379 26514841

[B26] Collins L. Collins D. (2022). The role of situational awareness in the professional judgment and decision-making of adventure sport coaches. J. Expertise 5, 117–131.

[B27] Cope E. Cushion C. J. Harvey S. Partington M. (2022). Re-visiting systematic observation: a pedagogical tool to support coach learning and development. Front. Sports Active Living 4:962690. 10.3389/fspor.2022.962690 36081620 PMC9446450

[B28] Curran T. Standage M. (2017). Psychological needs and the quality of student engagement in physical education: teachers as key facilitators. J. Teach. Phys. Educ. 36, 262–276. 10.1123/jtpe.2017-0065

[B29] DAgostino R. B. (2017). “Tests for the normal distribution,” in Goodness-of-Fit-Techniques, (London: Routledge).

[B30] das Neves Salles W. Martins L. C. do Nascimento J. V. Milistetd M. (2025). It's not always easy to buy the idea: strategies, perceptions, and implications of learner-centered teaching in coach education. Front. Sports Active Living 7:1534372. 10.3389/fspor.2025.1534372 39935711 PMC11810921

[B31] Deci E. L. Koestner R. Ryan R. M. (1999). A meta-analytic review of experiments examining the effects of extrinsic rewards on intrinsic motivation. Psychol. Bull. 125:627. 10.1037/0033-2909.125.6.627 10589297

[B32] Deci E. L. Ryan R. M. (1980). The empirical exploration of intrinsic motivational processes. Adv. Experim. Soc. Psychol. 13, 39–80. 10.1016/S0065-2601(08)60130-6

[B33] Deci E. L. Ryan R. M. (2008). Facilitating optimal motivation and psychological well-being across life's domains. Can. Psychol. 49:14. 10.1037/0708-5591.49.1.14

[B34] Deci E. L. Ryan R. M. (2013). Intrinsic Motivation and Self-Determination in Human Behavior. Cham: Springer Science & Business Media.

[B35] Demiral,¸ S. Nazıroğlu M. (2024). Examination of experienced coaches and physical education teachers' teaching methods and their perceptions regarding these methods–2023. Front. Sports Active Living 6:1383361. 10.3389/fspor.2024.1383361 38887685 PMC11180728

[B36] Deng J. Che T. Xiao C. Wang S. Dai L. Meerzhan A. (2019). Suitability analysis of ski areas in China: an integrated study based on natural and socioeconomic conditions. Cryosphere 13:2149–2167. 10.5194/tc-13-2149-2019

[B37] Dick A. S. Basu K. (1994). Customer loyalty: Toward an integrated conceptual framework. J. Acad. Market. Sci. 22, 99–113. 10.1177/009207039422200130382739

[B38] Eastabrook C. Collins L. (2021). What do participants perceive as the attributes of a good adventure sports coach? J. Advent. Educ. Outdoor Learn. 21, 115–128. 10.1080/14729679.2020.1730207

[B39] Ettl Rodríguez F. I. Falcão W. R. McCarthy J. (2023). Coach as youth development specialist: developing a tpsr-based coach training program and examining participants experiences. Phys. Educ. Sport Pedagogy. 2023, 1–24. 10.1080/17408989.2023.2281910

[B40] Fader P. S. Hardie B. G. Lee K. L. (2005). RFM and CLV: using iso-value curves for customer base analysis. J. Market. Res. 42, 415–430. 10.1509/jmkr.2005.42.4.41511670861

[B41] Falk M. Hagsten E. (2016). Importance of early snowfall for swedish ski resorts: Evidence based on monthly data. Tour. Managem. 53, 61–73. 10.1016/j.tourman.2015.09.002

[B42] Faullant R. Matzler K. Füller J. (2008). The impact of satisfaction and image on loyalty: the case of alpine ski resorts. Manag. Serv. Qual.: Int. J. 18, 163–178. 10.1108/09604520810859210

[B43] Galvan H. Fyall G. Culpan I. (2012). High-performance cricket coaches' perceptions of an educationally informed coach education programme. Asia-Pacific J. Health, Sport Phys. Educ. 3, 123–140. 10.1080/18377122.2012.700692

[B44] General Administration of Sport of China (2017). Professional Talent Gap Reaches 70%: *Ice and Snow Professionals in High Demand*. Beijing: General Administration of Sport of China.

[B45] General Administration of Sport of China (2024a). 2023 National Sports Venue Statistics and Survey Data. Beijing: General Administration of Sport of China.

[B46] General Administration of Sport of China (2024b). Research Report on the Mass Ice and Snow Consumption Market (2023-2024 Ice and Snow Season).

[B47] Gilakjani A. P (2012) Visual auditory, kinaesthetic learning styles and their impacts on english language teaching. Journal of studies in education 2: 104–113.35646326

[B48] Glasgow R. E. Riley W. T. (2013). Pragmatic measures: What they are and why we need them. Am. J. Prev. Med. 45, 237–243. 10.1016/j.amepre.2013.03.010 23867032

[B49] Gogtay N. J. Thatte U. M. (2017). Principles of correlation analysis. J. Assoc. Phys. India 65, 78–81. 28462548

[B50] Goodyear V. A. Casey A. Kirk D. (2013). Physical education teachers' use of practitioner inquiry: effective, enjoyable and relevant professional learning. Asia-Pacific J. Health, Sport Phys. Educ. 4, 19–33. 10.1080/18377122.2013.760425

[B51] Grant G. (2013). Short Blacks Or Flat Whites: Australian Snowsports; what Do Customers Want? A Study Into the Australian Snowsport Guest's Heterogeneity, Satisfaction, and Intention to Revisit and Recommend (PhD thesis). University of Canberra, Bruce ACT, Australia.

[B52] Gray S. Mitchell F. Wang C. J. Robertson A. (2018). Understanding students experiences in a pe, health and well-being context: a self-determination theory perspective. Curric. Stud. Health Phys. Educ. 9, 157–173. 10.1080/25742981.2018.1442230

[B53] Greene J. D. (1973). Repeat-buying: Theory and applications. JMR, J. Market. Res. 10:454.

[B54] Guadagnoli M. A. Lee T. D. (2004). Challenge point: A framework for conceptualizing the effects of various practice conditions in motor learning. J. Mot. Behav. 36, 212–224. 10.3200/JMBR.36.2.212-224 15130871

[B55] Han X. Jia L. (2023). Safety and prevention of sports injuries in popular ice and snow sports. Revista Brasileira de Medicina do Esporte 29:e2022. 10.1590/1517-8692202329012022_0747

[B56] Hasan M. K. Ismail A. R. Islam M. F. (2017). Tourist risk perceptions and revisit intention: a critical review of literature. Cogent Bus. Manage. 4:1412874. 10.1080/23311975.2017.1412874

[B57] Hattie J. Timperley H. (2007a). The power of feedback. Rev. Educ. Res. 77, 81–112. 10.3102/003465430298487Q1738293548

[B58] Hellier P. K. Geursen G. M. Carr R. A. Rickard J. A. (2003). Customer repurchase intention: a general structural equation model. Eur. J. Market. 37, 1762–1800. 10.1108/03090560310495456

[B59] Hennig-Thurau T. Langer M. F. Hansen U. (2001). Modeling and managing student loyalty: an approach based on the concept of relationship quality. J. Serv. Res. 3, 331–344. 10.1177/109467050134006

[B60] Hinkin T. R. Tracey J. B. Enz C. A. (1997). Scale construction: Developing reliable and valid measurement instruments. J. Hospital. Tour. Res. 21, 100-120. 10.1177/109634809702100108

[B61] Huang Y. Kim D. (2023). How does service quality improve consumer loyalty in sports fitness centers? The moderating role of sport involvement. Sustainability 15:12840. 10.3390/su151712840

[B62] Ibrahim R. H. Hussein D. A. (2016) Assessment of visual auditory, and kinesthetic learning style among undergraduate nursing students. Int J Adv Nurs Stud, 5 :1–4.

[B63] Ismert M. Petrick J. F. (2004). Indicators and standards of quality related to seasonal employment in the ski industry. J. Travel Res. 43, 46–56. 10.1177/0047287504265512

[B64] Jang H. Kim E. J. Reeve J. (2016). Why students become more engaged or more disengaged during the semester: a self-determination theory dual-process model. Learn. Instruct. 43, 27–38. 10.1016/j.learninstruc.2016.01.002

[B65] Jang H. Reeve J. Deci E. L. (2010a). Engaging students in learning activities: it is not autonomy support or structure but autonomy support and structure. J. Educ. Psychol. 102, 588. 10.1037/a0019682

[B66] Jiang Y.-y. Zhang Y. Gao J. Zhang Y.-y. Fang Y. (2022). High-quality development of ice and snow resources in China: theoretical review, practice turn and challenge response. J. Nat. Res. 37, 2334–2347. 10.31497/zrzyxb.20220910

[B67] Kiers H. A. (1997). Weighted least squares fitting using ordinary least squares algorithms. Psychometrika 62, 251–266. 10.1007/BF02295279

[B68] Klein J. P. Moeschberger M. L. Klein J. P. Moeschberger M. L. (2003). “Hypothesis testing,” in. *Survival Analysis: Techniques for Censored and Truncated Data* (New York, NY: Springer), 201–242. 10.1007/b97377

[B69] Kluger A. N. DeNisi A. (1996). The effects of feedback interventions on performance: a historical review, a meta-analysis, and a preliminary feedback intervention theory. Psychol. Bull. 119, 254. 10.1037/0033-2909.119.2.254

[B70] Koenker R. Hallock K. F. (2001). Quantile regression. J. Econ. Perspect. 15, 143–156. 10.1257/jep.15.4.143

[B71] Koh K. T. Camiré M. Regina S. H. L. Soon W. S. (2017). Implementation of a values training program in physical education and sport: a follow-up study. Phys. Educ. Sport Pedagogy 22, 197–211. 10.1080/17408989.2016.1165194

[B72] Koo M. Yang S.-W. (2025). Likert-type scale. Encyclopedia 5:18. 10.3390/encyclopedia5010018

[B73] Kranzinger C. Kranzinger S. Hollauf E. Rieser H. Stöggl T. (2024). Skiing quality analysis of recreational skiers based on imu data and self-assessment. Front. Sports Active Living 6:1495176. 10.3389/fspor.2024.1495176 39777018 PMC11703856

[B74] Kyle G. T. Theodorakis N. D. Karageorgiou A. Lafazani M. (2010). The effect of service quality on customer loyalty within the context of ski resorts. J. Park Recreat. Admi. 28, 1–15.

[B75] Leeder T. M. Beaumont L. C. (2025). Teaching and Coaching Lifestyle Sports: Research and Practice. London:L Routledge.

[B76] Lei F. Yue L. Zhandong Y. (2022). Influence of ice and snow sports participation experience on participation constraints among residents in Southern China: a quantitative analysis based on propensity score matching. J. Resour. Ecol. 13, 624–634. 10.5814/j.issn.1674-764x.2022.04.008

[B77] Li C. Jie L. (2022). How many Efforts are Needed to Ignite the “Ice and Snow Fever” in the South? Seoul: Economic Daily.

[B78] Li Y. Zhao M. Guo P. Zheng J. Li Z. Li F.. (2016). Comprehensive evaluation of ski resort development conditions in northern China. Chinese Geograp. Sci. 26, 401–409. 10.1007/s11769-016-0808-z

[B79] Li Z. Yuqin W. (2023). A Coach Hired for 20,000 Yuan Can't Even Put on Snow Boots Properly. Beijing: Legal Daily.

[B80] Liang L. Huang W. Huang S. Weng D. (2023). “Combining spssau and wjx. cn analysis to study the status quo of online and offline blended teaching model,” in Proceedings of the 2nd International Conference on Internet, Education and Information Technology (IEIT 2022). Cham: Springer Nature.

[B81] Light R. Curry C. Mooney A. (2014). Game sense as a model for delivering quality teaching in physical education. Asia-Pacific J. Health, Sport Phys.Educ. 5, 67–81. 10.1080/18377122.2014.868291

[B82] Lin Y. Aoyu L. Changyang X. Shuang L. (2024). Sichuan ski instructor teaching challenges and countermeasures research. *Sichuan Ski Instruct*. Teach. Challeng. Countermeas. Res. 6, 524–528. 10.35534/scps.0604053

[B83] Long L. Lijia L. (2024). What drives repurchase retention in music training institutions? Examining the roles of customer satisfaction, perceived value, and service quality. PLoS ONE 19:e0312087. 10.1371/journal.pone.0312087 39739734 PMC11687669

[B84] Ma X. Li J.-Y. Andd S. G. Ao Y.-F. Yang Y.-P. (2023). Comparison and analysis of skiing injuries at ski resorts in chongli, China and japan. Chin. J. Traumatol. 26, 63–67. 10.1016/j.cjtee.2022.08.002 36180308 PMC10071314

[B85] Meng X. Horrell A. McMillan P. (2025). “health first, safety first: an analysis of the legal system and professional ethics for curriculum enactment in China. Sport, Educ. Soc. 30, 42–56. 10.1080/13573322.2023.2283069

[B86] Miragaia D. Conde D. Soares J. (2016). Measuring service quality of ski resorts: An approach to identify the consumer profile. Open Sports Sci. J. 9:1. 10.2174/1875399X01609010053

[B87] Mittal V. Kamakura W. A. (2001). Satisfaction, repurchase intent, and repurchase behavior: Investigating the moderating effect of customer characteristics. J. Market. Res. 38, 131–142. 10.1509/jmkr.38.1.131.1883211670861

[B88] Mödinger M. Woll A. Wagner I. (2022). Video-based visual feedback to enhance motor learning in physical education–a systematic review. German J. Exerc. Sport Res. 52, 447–460. 10.1007/s12662-021-00782-y

[B89] Morales-Belando M. T. Côté J. Arias-Estero J. L. (2023). A longitudinal examination of the influence of winning or losing with motivational climate as a mediator on enjoyment, perceived competence, and intention to be physically active in youth basketball. Phys. Educ. Sport Pedagogy 28:568–581. 10.1080/17408989.2021.2006620

[B90] Moravecz M. Kovács K. E. Kozma B. (2025). Socialisation scenes in the health behaviour of teacher students at different levels of teacher training. Front. Sports Active Living 6:1504214. 10.3389/fspor.2024.1504214 39835189 PMC11743719

[B91] Müller M. Jürgens J. Redaèlli M. Klingberg K. Hautz W. E. Stock S. (2018). Impact of the communication and patient hand-off tool SBAR on patient safety: a systematic review. BMJ Open 8:e022202. 10.1136/bmjopen-2018-022202 30139905 PMC6112409

[B92] Neyman J. (1992). “On the two different aspects of the representative method: the method of stratified sampling and the method of purposive selection,” in Breakthroughs in Statistics: Methodology and Distribution (Cham: Springer), 123–150.

[B93] Nicol D. J. Macfarlane-Dick D. (2006). Formative assessment and self-regulated learning: A model and seven principles of good feedback practice. Stud. Higher Educ. 31, 199–218. 10.1080/03075070600572090

[B94] Pan S. J. (2020). Transfer learning. Learning 21, 1–2. 10.1017/9781139061773

[B95] Panadero E. (2023). Toward a paradigm shift in feedback research: Five further steps influenced by self-regulated learning theory. Educ. Psychol. 58, 193–204. 10.1080/00461520.2023.2223642

[B96] Peng J. Zhang Y. Sato S. (2024). “Sport nationalism in China: the origin, development trajectory, and trends after the 2022 beijing winter olympic games,” in Research Handbook on Major Sporting Events (Massachusetts, USA: Edward Elgar Publishing), 472–484.

[B97] Pighetti J. (2021). Professional Development and Education Engagement Among Outdoor Professionals: A Data Mining Case Study on Snowsports Instructors (Master's thesis). The Pennsylvania State University, University Park, PA, United States.

[B98] Professional Ski Instructors of America (PSIA) and American Association of Snowboard Instructors (AASI) (2022). Teaching Skills Performance Guide. Lakewood, CO: PSIA-AASI.

[B99] PSIA-AASI (2025). The Learning Connection & *Discipline-Specific Fundamentals*. Lakewood, CO: PSIA-AASI.

[B100] PSIA-AASI Western Division (2018). The New Teaching Snowsports Manual. Available online at: https://psia-w.org/the-new-teaching-snowsports-manual/ (Accessed 21 August, 2025).

[B101] Reeve J. Cheon S. H. (2021a). Autonomy-supportive teaching: Its malleability, benefits, and potential to improve educational practice. Educ. Psychol. 56, 54–77. 10.1080/00461520.2020.1862657

[B102] Renshaw I. Chow J. Y. (2019). A constraint-led approach to sport and physical education pedagogy. Phys. Educ. Sport Pedag. 24, 103–116. 10.1080/17408989.2018.1552676

[B103] Richards K. A. R. Gordon B. (2017). Socialisation and learning to teach using the teaching personal and social responsibility approach. Asia-Pacific J. Health, Sport Phys. Educ. 8, 19–38. 10.1080/18377122.2016.1272424

[B104] Rios-Avila F. Maroto M. L. (2024). Moving beyond linear regression: implementing and interpreting quantile regression models with fixed effects. Sociol. Methods Res. 53, 639–682. 10.1177/00491241211036165

[B105] Roberts P. Priest H. Traynor M. (2006). Reliability and validity in research. Nurs. Stand. 20:44. 10.7748/ns.20.44.41.s56 16872117

[B106] Rosenshine B. (2012). Principles of instruction: research-based strategies that all teachers should know. Am. Educ. 36:12. Available online at: https://files.eric.ed.gov/fulltext/EJ971753.pdf (Accessed September 3, 2025).

[B107] Ryan R. M. Deci E. L. (2000). Self-determination theory and the facilitation of intrinsic motivation, social development, and well-being. Am. Psychol. 55:68. 10.1037/0003-066X.55.1.68 11392867

[B108] Ryan R. M. Deci E. L. (2024). “Self-determination theory,” in Encyclopedia of Quality of Life and Well-Being Research (Cham: Springer), 6229–6235.

[B109] Salmoni A. W. Schmidt R. A. Walter C. B. (1984). Knowledge of results and motor learning: a review and critical reappraisal. Psychol. Bull. 95, 355–386. 10.1037/0033-2909.95.3.355 6399752

[B110] Senior Specialist Manual (2008). Senior Specialist Manual. Lakewood, CO: PSIA-AASI.

[B111] Shelley K. O'Hara L. Gregg J. (2010). The impact on teachers of designing and implementing a health at every size curriculum unit. Asia-Pacific J. Health, Sport Phys. Educ. 1, 21–28. 10.1080/18377122.2010.9730334

[B112] Shen P.-H. (2016). Relationships Among Service Quality, Motivations, and Revisit Intentions for Taiwanese Skiers and Snowboarders (Ph.d. dissertation). Middle Tennessee State University, Murfreesboro, Tennessee, United States.

[B113] Shute V. J. (2008). Focus on formative feedback. Rev. Educ. Res. 78, 153–189. 10.3102/003465430731379538293548

[B114] Sierens E. Vansteenkiste M. Goossens L. Soenens B. Dochy F. (2009). The synergistic relationship of perceived autonomy support and structure in the prediction of self-regulated learning. Br J. Educ. Psychol. 79, 57–68. 10.1348/000709908X304398 18466671

[B115] Siqi Z. Chaetnalao A. (2024). A new perspective on snowboarding: an in-depth exploration of beginner education experiences. J. Commu. Dev. Res. 17, 130–145. 10.14456/jcdr-hs.2024.8

[B116] Snow Operating L. L. C. (2015). Terrain Based Learning: Resort Expert Trainer Program Guide Book. Snow Operating.

[B117] Spittle M. Byrne K. (2009). The influence of sport education on student motivation in physical education. Physical education and sport pedagogy 14:253–266. 10.1080/17408980801995239

[B118] Steiger R. Scott D. Abegg B. Pons M. Aall C. (2019). A critical review of climate change risk for ski tourism. Curr. Issues Tour. 10.1080/13683500.2017.1410110

[B119] Stringfellow A. Wang C.-h. Farias C. F. Hastie P. A. (2024). The development of an “engagement in physical education” scale. Front. Sports Active Living 6:1460267. 10.3389/fspor.2024.1460267 39664742 PMC11631609

[B120] Sun K. Tian X. Xia J. Ou M. Tang C. (2023). The market responses of ice and snow destinations to southerners tourism willingness: a case study from China. Sustainability 15:13759. 10.3390/su151813759

[B121] Tang C.-c. Xiao X.-y. Han Y. Zeng R. Xu S.-y. Liu Y.-,r.. (2022). Development mode and optimization path of typical ice and snow tourism destinations in China. J. Nat. Resour. 37:2348–2366. 10.31497/zrzyxb.20220911

[B122] Teixeira D. S. Rodrigues F. Cid L. Monteiro D. (2022). Enjoyment as a predictor of exercise habit, intention to continue exercising, and exercise frequency: the intensity traits discrepancy moderation role. Front. Psychol. 13:780059. 10.3389/fpsyg.2022.780059 35250719 PMC8894246

[B123] Teixeira P. J. Carraça E. V. Markland D. Silva M. N. Ryan R. M. (2012). Exercise, physical activity, and self-determination theory: a systematic review. Int. J. Behav. Nutr. Phys. Activ. 9:78. 10.1186/1479-5868-9-78 22726453 PMC3441783

[B124] Thibaut E. Eakins J. Vos S. Scheerder J. (2017). Time and money expenditure in sports participation: The role of income in consuming the most practiced sports activities in flanders. Sport Management Review 20:455–467. 10.1016/j.smr.2016.12.002

[B125] Thomas R. M. Kobayashi V. N. (2014). Educational Technology-Its Creation, Development and Cross-Cultural Transfer. London: Elsevier.

[B126] Tide News (2023). Migratory “Snowbird” Coaches Flocking To Southern Ski Resorts: “*I Never Expected to be in Such High Demand*”. Hangzhou: Zhejiang Daily Newspaper Group.

[B127] Tobias S. Carlson J. E. (1969). Brief report: Bartlett's test of sphericity and chance findings in factor analysis. Multivariate Behav. Res. 4, 375–377. 10.1207/s15327906mbr0403_8 26745847

[B128] Tomlinson C. A. McTighe J. (2006). Integrating Differentiated Instruction & *Understanding by Design: Connecting Content and Kids*. Arlington, VA: ASCD.

[B129] Tuu H. H. Olsen S. O. (2010). Nonlinear effects between satisfaction and loyalty: An empirical study of different conceptual relationships. J. Target. Measurem. Analy. Market. 18, 239–251. 10.1057/jt.2010.19

[B130] van der Meer B. R. van den Hoven M. A. C. van der Kamp J. Savelsbergh G. J. P. (2024). Self-controlled video feedback facilitates the learning of tactical skills in tennis. Res. Quart. Exerc. Sport, 95, 537–545. 10.1080/02701367.2023.2275801 38100576 PMC11147453

[B131] Vansteenkiste M. Ryan R. M. (2013a). On psychological growth and vulnerability: basic psychological need satisfaction and need frustration as a unifying principle. J. Psychother. Integr. 23:263. 10.1037/a0032359

[B132] Wei Y. Li J. Luo D. Tang X. Wu Z. Wang X. (2025). Risks and sustainability of outdoor ski resorts in China under climate changes. NPJ Climate Atmosph. Sci. 8:26. 10.1038/s41612-025-00917-0

[B133] Weiner B. (1990). History of motivational research in education. J. Educ. Psychol. 82:616. 10.1037/0022-0663.82.4.616

[B134] Wicker P. Hallmann K. Breuer C. (2013). Analyzing the impact of sport infrastructure on sport participation using geo-coded data: Evidence from multi-level models. Sport Managem. Re. 16, 54–67. 10.1016/j.smr.2012.05.001

[B135] Wisniewski B. Zierer K. Hattie J. (2020). The power of feedback revisited: a meta-analysis of educational feedback research. Front. Psychol. 10:487662. 10.3389/fpsyg.2019.03087 32038429 PMC6987456

[B136] Wu X. Liu Z. (2020). Comparison on curriculum setting of winter sports in universities in China, america and europe. Open J. Soc. Sci. 8:529–539. 10.4236/jss.2020.83046

[B137] Wu Y.-C. Hsieh L.-F. Lu J.-J. (2015). What's the relationship between learning satisfaction and continuing learning intention? Procedia-Soc. Behav. Sci. 191, 2849–2854. 10.1016/j.sbspro.2015.04.14827409075

[B138] Wulf G. (2013). Attentional focus and motor learning: A review of 15 years. Int. Rev. Sport Exerc. Psychol. 6, 77–104. 10.1080/1750984X.2012.723728

[B139] Yin S. Tang F. Gao P. (2025). Development and validation of the physical fitness test anxiety scale for college students. Front. Psychol. 16:1573530. 10.3389/fpsyg.2025.1573530 40290542 PMC12023481

[B140] Yoshida M. Sato M. Pizzo A. D. Kuramasu R. (2023). The evolution of psychological involvement and customer loyalty: a longitudinal analysis of fitness facility members. Sport Managem. Rev. 26, 744–765. 10.1080/14413523.2023.2215557

[B141] Yu C. Kong S. Yang H. Li Y. (2024). Development of ice and snow tourism destinations in China: ecological security assessment and obstacle degree analysis. Curr. Issues Tour. 2024, 1–22. 10.1080/13683500.2024.2345818

[B142] Zhang S. Chaetnalao A. (2024). Theoretical analysis of teaching models for snowboarding beginners. J Pacific Instit. Managem. Sci. 10, 298–312. Available online at: https://so05.tci-thaijo.org/index.php/pacific/article/view/274695/185169

[B143] Zhang W. Li H. Wang D. Xu G. Xu C. Li J.. (2023). The global research status and trends in ice and snow sports injuries from 1995 to 2022: A bibliometric and visualized analysis. Int. J. Environ. Res. Public Health 20:2880. 10.3390/ijerph20042880 36833576 PMC9957478

[B144] Zhou X. Zhang M. (2024). “Case study on sg performance rand uity rand oe and rand espect in a south korean ski esort,” in Prace Komisji Geografii Przemyslu Polskiego Towarzystwa Geograficznego. [Polish].

