# Supplementary material for: Associations between snowboard coaches teaching interaction, motion guidance, and effectiveness assessment competencies and learners intentions to continue lessons
Source: Front Psychol. 2025 Sep 23;16:1633094. doi: 10.3389/fpsyg.2025.1633094 (PMC12500609; doi:10.3389/fpsyg.2025.1633094)
Supplement: Supplementary file 1 [file Data_Sheet_1.pdf]

Survey on the Optimization of Snowboarding Courses in China

Dear Participants and Guardians,

Greetings! Thank you for your time participating in this survey. This study explores the relationship between snowboard instructors' teaching behaviors and competencies,aiming to further optimize and enhance China's snowboarding curriculum content.The questionnaire comprises six sections with 26 questions, taking approximately 5–8 minutes. Conducted through offline invitations and online responses, it is anonymous—no personal identification will be collected. All data remains strictly confidential for academic research purposes only.

0.Have you read the above information and consented to participate in this anonymous survey?

A. I am 18 years or older, have read the information, and consent to participate.
B. I am under 18, and have obtained informed consent from my legal guardian to participate

Basic Information

1.What is your age?

2.What is your gender?
A.Male
B. Female

3.How long have you been learning to ski?
A. Less than one ski season
B.One to three ski seasons
C. More than three seasons

4. What is your preferred learning style? [Multiple-choice question]
A. Visual (e.g., diagrams, videos, reading materials)
B. Auditory (e.g., lectures, discussions)
C. Kinesthetic (e.g., hands-on practice)

Interpersonal Skills

Please rate the following descriptions:

5. How satisfied are you with the instructor's attentiveness to your questions and suggestions during this course?

A. Very satisfied

B. Satisfied

C. Neutral

D. Unsatisfied

E.Very disappointed

6. How satisfied are you with the instructor's use of two-way communication to collaboratively set learning goals in this course?

A. Very satisfied

B. Satisfied

C. Neutral

D. Unsatisfied

E.Very disappointed

7. How satisfied are you with the instructor's reflective teaching approach to improve the learning experience in this course?

A.Very satisfied

B. Satisfied

C. Neutral

D. Unsatisfied

E. Very disappointed

8. How satisfied are you with the instructor's methods for motivating students and building confidence in this course?

A.Very satisfied

B.Satisfied

C.Neutral

D.Unsatisfied

E.Very disappointed

Interpersonal Skills

Please rate the following descriptions:

9. How satisfied are you with the instruction on executing smoother turns in this course?

A.Very satisfied

B.Satisfied

C.Neutral

D.Unsatisfied

E.Very disappointed

10. How satisfied are you with the instruction on skating/skate walking techniques (including slow one-foot gliding and back foot braking) in this course?

A. Very satisfied

B. Satisfied

C. Neutral

D. Unsatisfied

E.Very disappointed

11. How satisfied are you with the instruction on safe falling techniques (e.g., methods to reduce injury risks) in this course?

A. Very satisfied

B. Satisfied

C. Neutral

D. Unsatisfied

E.Very disappointed

12. How satisfied are you with the instruction on lift-riding skills (e.g., one-foot turns and chairlift boarding techniques) in this course?

A.Very satisfied

B.Satisfied

C.Neutral

D.Unsatisfied

E.Very disappointed

Instructional Skills

Please rate the following descriptions:

12. How satisfied are you with the instructor's ability to identify movement issues through observing your skiing/riding?

A. Very satisfied

B. Satisfied

C. Neutral

D. Unsatisfied

E.Very disappointed

14. How satisfied are you with the instructor's use of creative activities/games to enhance learning engagement?

A.Very satisfied

B.Satisfied

C.Neutral

D.Unsatisfied

E.Very disappointed

15. How satisfied are you with the instructor's provision of effective guidance based on analysis of your movement results?

A. Very satisfied

B. Satisfied

C. Neutral

D. Unsatisfied

E.Very disappointed

16. How satisfied are you with the instructor's development of improvement plans through movement problem assessment?

A. Very satisfied

B. Satisfied

C. Neutral

D. Unsatisfied

E.Very disappointed

17. How satisfied are you with the instructor's effective instruction delivery based on movement analysis outcomes?

A. Very satisfied

B. Satisfied

C. Neutral

D. Unsatisfied

E.Very disappointed

18. How satisfied are you with the instructor's implementation of the complete "Observe-Assess-Instruct" cycle for movement analysis?

A.Very satisfied

B.Satisfied

C.Neutral

D.Unsatisfied

E.Very disappointed

Overall Satisfaction

Please rate the following description:

19. How satisfied are you with the instructor’s overall performance (including interpersonal skills, technical expertise, and instructional competency) in this course?

Answer options:

A. Very satisfied

B. Satisfied

C. Neutral

D. Unsatisfied

E.Very disappointed

Experience Loyalty Dimensions

Please rate the following descriptions:

20. If the snowboarding course is optimized per the proposed improvements (e.g., enhanced interpersonal skills, technical expertise, instructional competency), would you still choose to enroll?

A. Strongly agree

B. Agree

C. Neutral

D. Disagree

E. Strongly disagree

21. If optimized per the above directions, would you sign up for advanced courses from this program in the near future?

A. Strongly agree

B. Agree

C. Neutral

D. Disagree

E. Strongly disagree

22. If optimized per these improvements, would you recommend this course to friends?

A. Strongly agree

B. Agree

C. Neutral

D. Disagree

E. Strongly disagree
