# Supplementary material for: Associations between snowboard coaches teaching interaction, motion guidance, and effectiveness assessment competencies and learners intentions to continue lessons
Source: Front Psychol. 2025 Sep 23;16:1633094. doi: 10.3389/fpsyg.2025.1633094 (PMC12500609; doi:10.3389/fpsyg.2025.1633094)
Supplement: Supplementary file 2 [file Data_Sheet_2.pdf]

1 The Three Rotated Dimensions

Table 1: Survey Questions by Rotated Dimensions

**Category**	**Question**
**Teaching Interaction and Feedback (TIF)**	5. Are you satisfied with the coach's listening and response?<br>6. Are you satisfied with the coach's discussion of learning goals?<br>7. Are you satisfied with the coach's reflection on teaching methods?<br>8. Are you satisfied with the coach's ability to motivate learners?
**Motion Guidance and Optimization (MGO)**	9. Are you satisfied with the instruction on turning techniques?<br>10. Are you satisfied with one-footed snowboarding instruction?<br>11. Are you satisfied with the instruction on falling techniques?<br>12. Are you satisfied with the instruction on using chairlifts?<br>13. Are you satisfied with the coach's ability to observe and identify issues?<br>15. Are you satisfied with the coach's feedback and guidance?<br>17. Are you satisfied with guidance based on movement analysis?
**Effectiveness Assessment and Customization (EAC)**	14. Are you satisfied with learning activities and games?<br>16. Are you satisfied with movement assessment and improvement plans?<br>18. Are you satisfied with the "observe–assess–guide" process?

2 Additional Figures

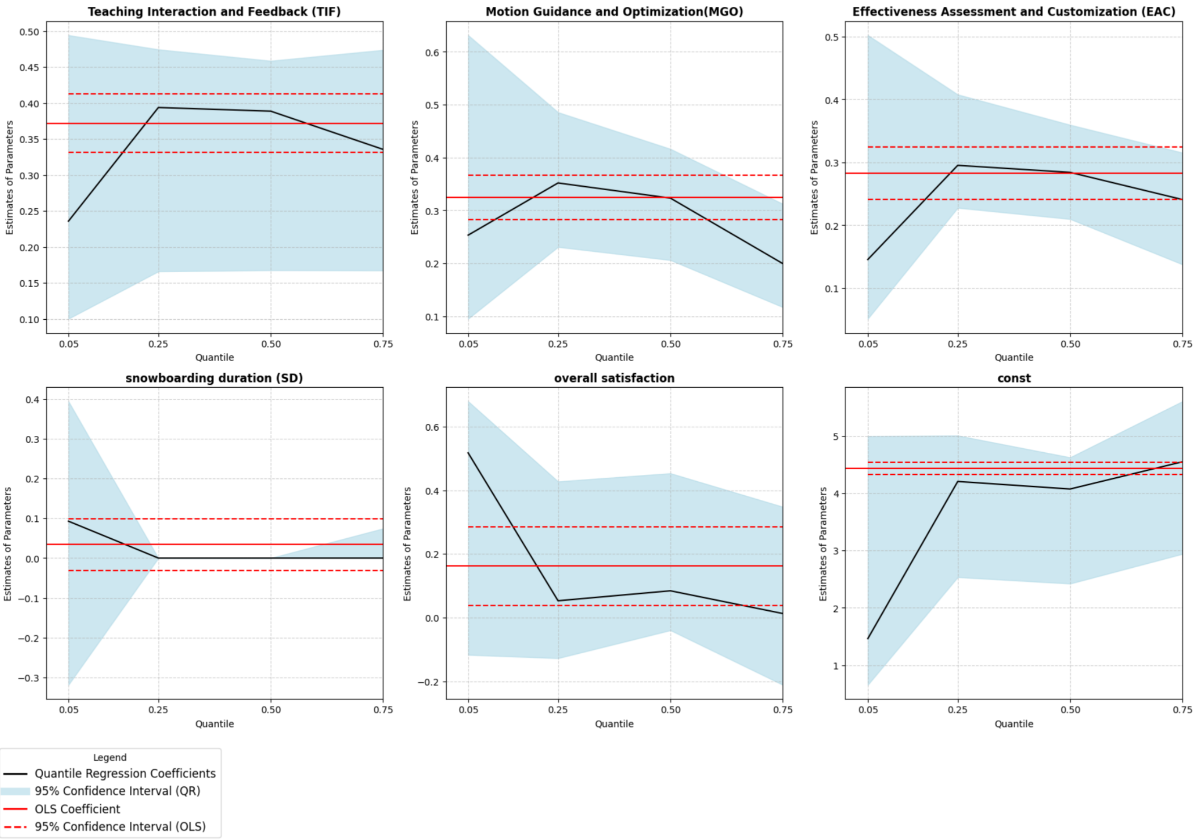

Figure 1: Quantile regression coefficients across loyalty quantiles. Black line: quantile-specific coefficients; blue band: 95% CI (quantile estimates); red solid: OLS coefficient; red dashed: 95% CI (OLS).
